# Interactive Sonification Exploring Emergent Behavior Applying Models for Biological Information and Listening

**DOI:** 10.3389/fnins.2018.00197

**Published:** 2018-04-27

**Authors:** Insook Choi

**Affiliations:** Studio for International Media & Technology, MediaCityUK, School of Arts & Media, University of Salford, Manchester, United Kingdom

**Keywords:** sonification, listening, emergent behavior, interaction design, cognitive cycle, supramodal attention, biological information, media psychology

## Abstract

Sonification is an open-ended design task to construct sound informing a listener of data. Understanding application context is critical for shaping design requirements for data translation into sound. Sonification requires methodology to maintain reproducibility when data sources exhibit non-linear properties of self-organization and emergent behavior. This research formalizes interactive sonification in an extensible model to support reproducibility when data exhibits emergent behavior. In the absence of sonification theory, extensibility demonstrates relevant methods across case studies. The interactive sonification framework foregrounds three factors: reproducible system implementation for generating sonification; interactive mechanisms enhancing a listener's multisensory observations; and reproducible data from models that characterize emergent behavior. Supramodal attention research suggests interactive exploration with auditory feedback can generate context for recognizing irregular patterns and transient dynamics. The sonification framework provides circular causality as a signal pathway for modeling a listener interacting with emergent behavior. The extensible sonification model adopts a data acquisition pathway to formalize functional symmetry across three subsystems: Experimental Data Source, Sound Generation, and Guided Exploration. To differentiate time criticality and dimensionality of emerging dynamics, *tuning functions* are applied between subsystems to maintain scale and symmetry of concurrent processes and temporal dynamics. Tuning functions accommodate sonification design strategies that yield order parameter values to render emerging patterns discoverable as well as *rehearsable*, to reproduce desired instances for clinical listeners. Case studies are implemented with two computational models, Chua's circuit and Swarm Chemistry social agent simulation, generating data in real-time that exhibits emergent behavior. *Heuristic Listening* is introduced as an informal model of a listener's clinical attention to data sonification through multisensory interaction in a context of structured inquiry. Three methods are introduced to assess the proposed sonification framework: Listening Scenario classification, data flow Attunement, and Sonification Design Patterns to classify sound control. Case study implementations are assessed against these methods comparing levels of abstraction between experimental data and sound generation. Outcomes demonstrate the framework performance as a reference model for representing experimental implementations, also for identifying common sonification structures having different experimental implementations, identifying common functions implemented in different subsystems, and comparing impact of affordances across multiple implementations of listening scenarios.

## Introduction: what do we listen to when we listen to data?

Sonification is an open-ended design task. Methods differ based on applications. Understanding the application context is critical for shaping listening scenarios and design requirements and subsequent choice of data translation strategies and sound production. In cases where the experimental data source is predictable in terms of well-defined data dimensions and boundaries, the relationship between system parameters and sound parameters can be relatively linear. However, when the experimental data source is unpredictable and the data exhibits emergent behavior, sonification requires a methodology to establish reliable rendition of the dynamics.

In the absence of established sonification theory, the field is challenged by *ad-hoc* variance in instruments, implementations and interpretations, limiting the scalability of case study results. This research examines the rationale and feasibility to formalize an extensible model for interactive sonification, applied to data that exhibits emergent behavior. An extensible model is proposed as an interactive sonification framework foregrouding three factors: reproducible system implementation for generating sonification, reproducible data from models that characterize emergent behavior, and interactive mechanisms enhancing a listener's multisensory observations. Using auditory sensation to seek biological information dates back to ancient Greek times when physicians monitored pulses for a clinical diagnosis (Kaniusas, 2012). The importance of engaging all senses—to see, feel, and hear—was emphasized in order to recognize patterns and put them to use (Castiglioni, 1947). Diagnoses by listening, termed *auscultation* from the nineteenth century (Laennec and Forbes, 1835), is limited to natural signals exhibiting amplitude and frequency accessible to human hearing. Patients are observed through multisensory interaction including touch and visual inspection (see Appendix 1 in Supplementary Material). The work presented here maintains a multisensory and multimodal approach in configuring sound to convey signatures of non-linear behavior that are characteristic of biological information. Today the auditory monitoring of physiological states is extended with modern equipment and digital signal processing inevitably introducing layers of artifacts. The very focus, or rather the intent of the motivation to listen to biological information when working with extended instrumentation and digital abstraction, is what this presentation aims to be in service of.

**Figure 1:**
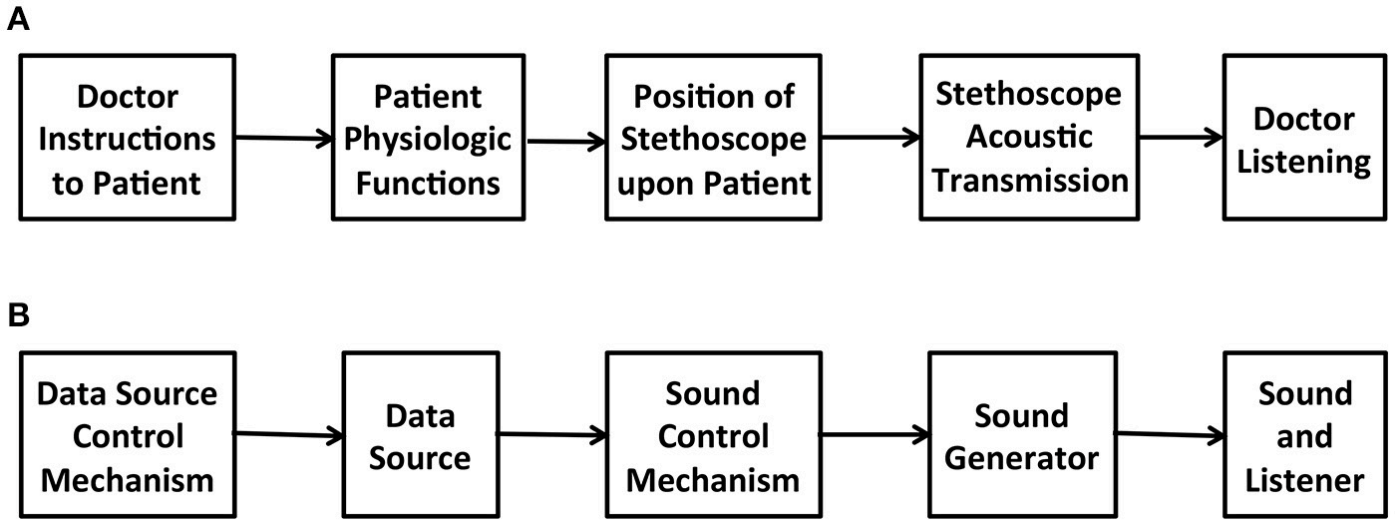
**(A)** Auscultation functional pathway including physician interaction with patient to induce physiological changes that generate audible differences. **(B)** Interactive sonification functional pathway comprised of requisite system components. The illustration shows parallel structure to auscultation **(A)**.

Experimental observation uses various methodologies to obtain information from a data source external to the observing system. To make sense of information the observing system performs measurements in order to gain insights about the states of the observed data source. Perceptualization implies transformation of data to yield observable information. To interpret data it is necessary to understand the transformations that bring the data into an observable form. “The algorithms that transform data are at the heart of data visualization.” (Hansen and Johnson, 2005, p. 1). Observers acquire the ability to interpret data by becoming familiar with the instruments that transform data, for example a doctor performs auscultation by understanding the functional attributes of the stethoscope, which informs interpretation of the transmitted sounds. Figure 1A illustrates the auscultation functional pathway. Sonification applies transformations requiring digital signal processing of machine-readable information, a functional pathway illustrated in Figure 1B, generalizing and digitizing the auscultation pathway. Since no pathway from information to data and back to information is immune to idiosyncratic complexity, the simplest definition is: *Sonification is a construction of sound that informs a listener of data*. In concept, sonification informed by biological data returns sounds that carry information about that biological system.

The present research aims to develop and extend sonification methods for data that exhibits emergent behaviors, addressing cases where reproducibility of covariance is quasi-deterministic for sounds and a corresponding data source. The research examines the viability of using models of emergent behavior to develop sonification methods that may be applied to multiple cases of biological information. Results presented here provide an example of using data models to formalize a sonification method to enable application with more than one type of data and more than one type of sound production. Following are assumptions based on sonification methods adopted in this research.

1. Sounds are audible because acoustic energy changes over time. Sound energy varies in multiple audible attributes, described in terms such as pitch, loudness, tone quality, duration, silence, repetition, and pattern. Depending upon the type of sound production, the number of audible attributes may be different than the number of sound control parameters required for those attributes.
2. Sonification aims for variance in sound to correspond to variance in the data represented by the sound. To sonify variance of data values requires a coherent model for covariance of sound control parameters. The goal is to enable a listener to identify salient features in sounds and relate these to salient features in the data.
3. The coupling of data to sound production requires four multidimensional component signals: the sound, having *m* audible attributes; the sound control signal, having *n* control parameters; the data to be sonified, having *p* dimensions; and the control signal applied to the data source, having *q* control parameters. These component signals have independent numbers of dimensions determined by the types of data, sound production, and observer interaction.

To establish a method for extensibility of results the coupling of sonification component signals is defined as a sonification model comprised of a series of functions that meet the following requirements:

- the sonification model is functional for multiple data sources, types of sound production, and modes of observer interaction;
- the model maintains its structure while the dimensionality of the component signals may change;
- the model is extensible to many applications as a sonification framework.

The present research applies a candidate framework to sonify two models of data sources that exhibit emergent behavior, Chua's circuit and Swarm Chemistry.

Biological systems are complex dynamical systems that may exhibit emergent behavior. Emergent behavior produces salient features in data that can vary independently of the control state of the data source. When applying sonification to a data source that exhibits emergent behavior, the aggregate coupling of sonification components may produce inconsistent correspondence of data features and sound features. To develop robust correspondence of sound to data, this research adopts data sources comprised of models that exhibit emergent behavior. Two sonification methods are presented here, one using stable regions in the data source to generate bounding reference sounds for unstable emergent regions, the other using automated feature recognition.

The use of models of emergent behavior for sonification test cases is adopted from research practices for measuring biological signals. When biological information is acquired experimentally, computational models are often used to ensure the relevance of the data and provide quality assurance for unstable and transient experimental conditions (James and Hesse, 2005). Simulations aid discernment and interpretation of transduced data, providing stable reference measurements for developing models of experimental physiological states. Interpreting neurological impulse patterns, Faure and Korn report, “The methods used in each of these studies have almost invariably combined the analysis of experimental data with simulations using formal models.” (Faure and Korn, 2003, p. 787). Biosignals can be modeled in terms of signature dynamic properties, for example simulation of spiking and bursting of cortical neurons (Izhikevich, 2003). Liljenström applies simulation of non-linear circuit oscillations to reproduce non-linear dynamics measured in brain signals, demonstrating chaotic oscillations as highly efficient for neural information processing. Simulations can be measured to demonstrate high sensitivity to input stimulus and rapid convergence on stable oscillations that may represent learned patterns (Liljenström, 2010, 2012).

In line with the use of computational models in experimental observation, the work reported here was performed with simulations recognized as paradigms for modeling biological signals. The rationale for selecting test cases is to identify models with properties that represent a broad range of applications. Emergent behaviors create non-deterministic conditions for sonification covariance with data pattern formation. Unpredictability limits the reliability of salient features' correspondence in data and sound. To address this the research applies models of data sources that exhibit emergent pattern formation. Wiener characterizes a pattern as an arrangement of elements, where the distinctness of the pattern is characterized by the order among the elements rather than the intrinsic qualities of individual elements (Wiener, 1954). This relationship can be quantitatively expressed as a set of higher-level order parameters, the concept introduced by Haken, which describes enslaving the behaviors of ensembles of elements by which patterns are formed (Haken, 1983). Biological systems in diverse areas of study have been observed to exhibit such ensembles' emergent properties. Examples are interaction patterns of groups of neurons expressed in the patterns of bursting (Wang and Rinzel, 1998), voltage oscillations in muscle fibers (Morris and Lecar, 1981), the patterns of clusters of autonomous agents at multicellular level such as behaviors demonstrated in Globally Coupled Maps (Kaneko, 2015) and multi-organism levels such as swarming and flocking behaviors of insects and birds (Charbonneau et al., 2013). These classes of examples suggest two paradigmatic models: oscillation and agents. These two levels of abstraction cover a wide range of cases for sonification of emergent behavior. The case of Chua's circuit is selected to demonstrate the oscillation model. Chua's circuit is a well-studied paradigm of non-linear dynamics (Chua, 2005), as it exhibits signal properties from periodic to chaotic as observed in many organisms including the brains of vertebrates and humans. The case of Swarm Chemistry is selected to demonstrate the agent model. Swarm Chemistry is an interactive evolutionary computing (IEC) framework for studying collective behaviors of self-organizational agents implemented as heterogeneous swarm simulation (Sayama, 2007). Chua's circuit and Swarm Chemistry exhibit dominant multi-paradigms of non-linear behaviors and yield emergent characteristics representative of biological information. In neurosciences, “Overall, both theoretical and experimental works in the field seem to demonstrate that the advanced tools of non-linear analysis can much more accurately describe and represent the complexity of brain dynamics than traditional mathematical and computational methods based on linear and deterministic analysis (Mattei, 2014, p. 1).” Incorporating the breadth of these paradigms, an adaptable sonification framework can be assessed for capacity to generate sounds that represent non-deterministic properties such as emergent features and patterns.

Perceptualization of data requires assumptions about observers that may be formalized as a model of an observer. Listener interaction with the data source provides a context for an informal model of an observer in a sonification framework. Interaction provides a frame of reference for a listener to identify emergent behavior by comparing changes in the state of the data source and behaviors exhibited in the data. A listener interacting with a data source can determine whether observed behaviors are emergent properties or are controllable by direct manipulation of the data source. Emergent behavior is more difficult to disambiguate if a listener is not interacting with the data source during observation. Models of observer interaction are required to support extensible outcomes of user assessment of sonification test cases. The hypothesized sonification framework includes a normalized representation of observer interaction assessed across multiple applications.

## Materials and models for interactive sonification of emergent behavior

This research applies an experimental configuration for user interaction and models implemented as computational simulations. Section Two Dynamical Systems Models: Commonalities and Differences introduces Chua's circuit and Swarm Chemistry, two computational models implemented as real-time applications with interactive control, to generate sample data that exhibits emergent behavior. A model of a data acquisition pathway is introduced in section Data Acquisition Model for Sonification Signal Processing, and applied in section Extended Model for a Sonification Framework for testing the hypothesis of extensible sonification modeling. In section Heuristic Listening and the LIDA Model the LIDA model is consulted to develop criteria accounting for a listener's disposition toward interactive sonification.

Physical materials required for this research include an instrument configuration for use case trials. The experimental configuration provides sound synthesis, two-channel stereo audio display, interface devices including computer mouse and large-format touch screen, computer graphic display of graphical user interfaces, and large-format display of data visualization. Case studies apply real-time interactive simulations in multisensory configurations. Sonification and visualization are synchronized with user generated interactive signals.

Sonification components are implemented as three subsystems for concurrent asynchronous processing: a data source to be rendered in sound, a sound production subsystem, and an observer interface for interactive exploration. Data transmission occurs at 10–20 Hz for user generated interactive signals and system control signals; visual display is refreshed at 24 Hz and sound is generated at 44.1 kHz per stereo channel. A scheduler ensures concurrent real-time responses across asynchronous processes.

### Two dynamical systems models: commonalities and differences

A dynamical system is iterative based on a numerical model that defines state, initial conditions and system control parameters. The simulation outputs data as a time series signal exhibiting dynamical system behavior defined as trajectories in a multidimensional phase space. The phase space represents all possible signal states. Control parameters define all possible states in control space. Control parameter values determine system conditions but may not directly determine system outputs. Complex dynamical systems exhibit self-organizing and emergent behaviors that are not predictable from previous states or from control parameter values (Silva, 1993).

Webber and Zbilut state that physiological systems can be best characterized as complex dynamical processes (Webber and Zbilut, 1994). They apply the lessons learned from complex systems theory that simple structure from a low dimensional network may generate a wide range of patterns with little experimental preparation. Chua's circuit and Swarm Chemistry exhibit this property. Chua's circuit has seven control parameters and Swarm Chemistry has six behavioral parameters. Both models exhibit emergent properties at multiple time scales. Salient features recur at periodic and aperiodic intervals, with short patterns sometimes embedded in long patterns. The Chua's circuit is a non-linear oscillator that generates a continuous-time signal from seven circuit components. Swarm Chemistry animates movements of hundreds of self-propelled agents along individual paths, defined in a bounded plane. For sound production Chua's circuit signal can be scaled to a human audible range whereas the Swarm Chemistry data comprises points in space that cannot directly produce sound. This contrast differentiates requirements for two approaches to sonification and offers insights to establishing a common framework. In both cases the sonification couples simulation dynamics with sound generation for listeners' exploratory interaction. Section Results: Applying the Proposed Sonification Framework to Multiple Experimental Systems presents differences in sonification design corresponding to structural differences of the simulations.

### Data acquisition model for sonification signal processing

**Figure 2:**
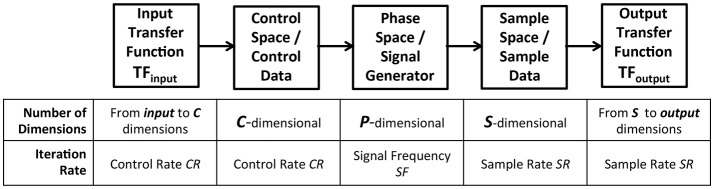
Data-elicitation pathway applied as a model to formalize the functions of subsystems required for sonification. Each component in the pathway is dynamic, receives input data, and generates output data. The data of each component may be represented by a unique number of dimensions. Each component is dynamic with a periodic iteration at one of three frequencies: a control rate, a signal frequency and a sample rate. TF_*input*_ receives data in *input* dimensions and generates *c*-dimensional data required for the control signal. Control space defines *c* dimensions for system control with minimum and maximum bounding values on each dimension, defining all possible states of control for signal generation. Phase space is multidimensional with *p* dimensions for system variables specifying the instantaneous state of the system output. Phase space encompasses all possible states of the output signal. Sample space represents a parameterized multivariate stream of digital data that discretizes the phase space signal. Sample space represents the signal in discrete time steps with a set of *s* values at each step; the sampling method and data format vary with each subsystem. TF_*output*_ receives the sample data in *s* dimensions and generates *output* dimensional data required downstream. Control space, phase space, and sample space define periodic iterations, indicated as control rate *CR*, signal frequency *SF*, and sample rate *SR*. Frequencies of these three periods may vary independently and are concurrent within the model. For example Chua's circuit has a control rate of 15–20 Hz, signal frequency rate of 20 kHz, and data sample rate of 44.1 kHz, while Swarm Chemistry has a control rate of 10 Hz, signal frequency rate of 20 Hz, and data sample rate of 12–15 Hz.

To test the hypothesis of extensible sonification modeling, a common model of data acquisition pathway is adopted for all sonfication component signals and applied to all use cases tested. The model is based on established practice and given rigorous uniform application to test the hypothesis that *a model can identify symmetry of functional requirements to represent all sonification components*. The data acquisition model, illustrated in Figure 2 provides five processes that connect control data to a signal generator and then to sample data, and perform data stream conversion from control rate iteration, to signal frequency, to sample rate iteration. The model in Figure 2 provides a basic and extensible organization.

- At the center of the data acquisition model a signal generator provides a source of sample data.
- Control data enters the model through a multidimensional transfer function TF_*input*_ and is applied to a set of parameters that control the signal generator.
- The resulting signal is sampled to acquire data that characterizes the signal, and this data is passed to a multidimensional transfer function TF_*output*_.

This design exposes and formalizes the functional requirements for managing differences in data dimensions and in temporal definition, which may occur between control signals and sampling processes. Frequency differences may result in serial oversampling or undersampling between processes, requiring adjustment to eliminate aliasing. Collectively these frequencies determine the overall frequency required for input data to affect output data in an implementation of the model. Sonification modeling formalizes over- and under-sampling differences among its temporal dynamics. Formal definition and systematic management of data dimensions and temporal differences are required to establish extensibility of sonification models to multiple application domains.

### Extended model for a sonification framework

**Figure 3:**
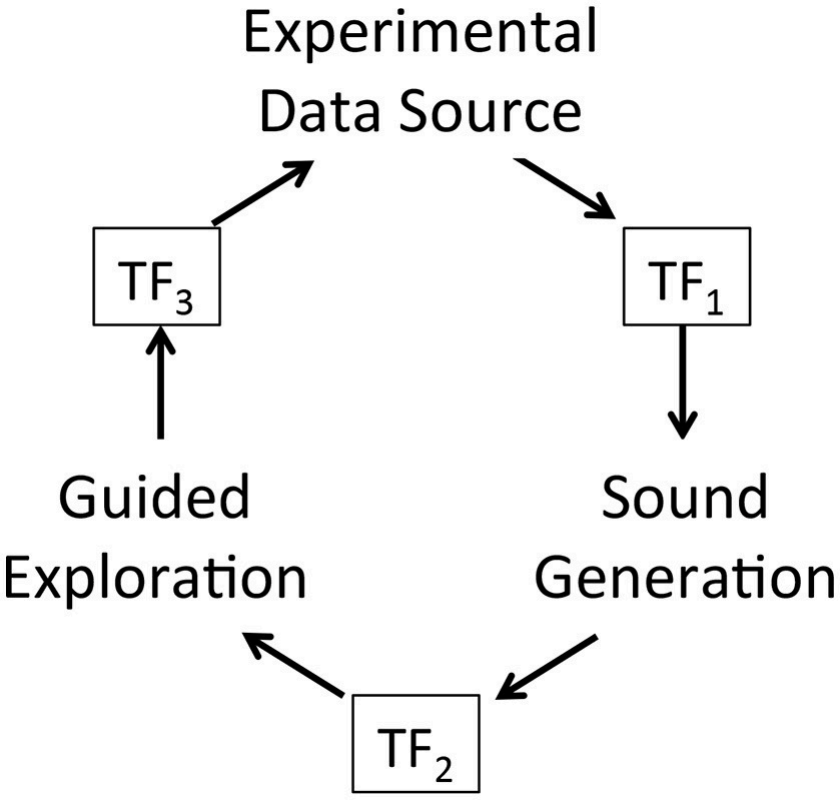
Proposed framework for interactive sonification based on an extended model of data acquisition. Three subsystems provide core functions required for interactive sonification. Three transfer functions define a data transmission pathway that enables circular causality. The extensibility of the data acquisition model establishes structural symmetry of the three subsystems.

A key research hypothesis is to establish an extensible sonification model. To implement this hypothesis the data acquisition model introduced above is adopted threefold to formalize each of three sonification subsystems: data source, sound production, and observer interface for interactive exploration. The model represents concurrent processes within a subsystem and by extension concurrent processes across the full sonfication implementation. The three subsystems are referred to as Experimental Data Source, Sound Generation, and Guided Exploration. In the proposed sonification model the subsystems are arranged in series with the output of one applied to the input of the next: output of Experimental Data Source is input to Sound Generation, the output of which is input to Guided Exploration, the output of which is input to control the Experimental Data Source. The iterative and concurrent nature of all processes can be represented as circularity of inputs and outputs, illustrated in Figure 3. Using the data acquisition reference model, a transfer function TF_*n*_ applies to each subsystem input and output. In circularity, each subsystem output TF_*n*_ performs a duplicate transfer function as the input of the next subsystem in the series.

**Figure 4:**
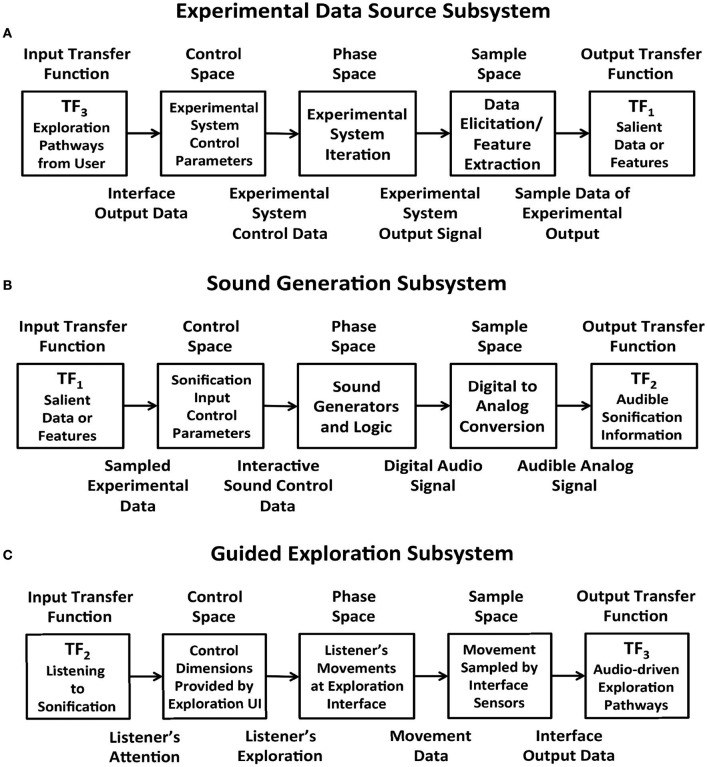
**(A)** Experimental Data Source subsystem represented using the data elicitation model. Data from a user interface enters through transfer function TF_3_ and is applied to the control parameters of a dynamic experimental system, which generates a stream of output data that exhibits emergent behavior. Characteristics of the signal are sampled to acquire relevant features and the sample stream is output to TF_1_. **(B)** Sound Generation subsystem represented using the data elicitation model. Data entering from TF_1_ is applied to the sound control parameters for sound generators. Associated sound design logic encodes procedures that generate sonification. The resulting digital audio signals are sampled to generate audible information output to TF_2_. **(C)** Guided Exploration subsystem represented using the data elicitation model. A listener positioned at a control interface attends to sonification input at TF_2._ The listener performs movements in response to the sonification, by manipulating the interface. In sample space the interface states from listener's actions are digitally acquired. At TF_3_ the interface samples are output to the control space of the experimental data source (see **panel A**). This enables the listener to explore the relationship between actions and audible changes that may be generated.

Figures 4A–C apply the data acquisition model to the particulars of each subsystem. In the Experimental Data Source subsystem (Figure 4A) data entering from TF_3_ is applied to the control space of Chua's circuit or Swarm Chemistry. The phase space exhibits stable states, transition states and emergent behaviors, which are sampled to acquire relevant features. The sample space is output to TF_1_. In the Sound Generation subsystem (Figure 4B) data entering from TF_1_ is applied to the sound control space, generating digital audio signals that are sampled to generate audible information output to TF_2_. In the Guided Exploration subsystem (Figure 4C) a listener acquires sound represented at TF_2_, and responds by manipulating an interface to explore the system through listening. The exploration signal is sampled and output to TF_3_, to be applied to the control space of the Experimental Data Source (Figure 4A).

To include a listener model, TF_2_ represents heuristic analysis rather than a formal computational model of listening. A listener performs heuristic analysis of audible signals received at TF_2_ to identify expected features that represent known data states, transformations and deviations. The term *heuristic listening* is introduced to describe a cognitive process that connects a listener's auditory percept to expectations and action planning (section Heuristic Listening and the LIDA Model).

To summarize the extended sonification model: circular causality is provided through three component subsystems that collectively form a round trip of data elicitation, audible interpretation, and listener response (Figure 3) Each subsystem is defined as a data acquisition pathway consisting of control space, phase space, and sample space. The data acquisition reference model recognizes functional tripartite symmetry of the subsystems. The data types passing through each subsystem are different but their data pathways share common functional relationships for generating, acquiring and interpreting data. This extended model is proposed as an Interactive Sonification Framework.

### Heuristic listening and the LIDA model

A model of listeners' engagement with sound provides essential context in the production of sound to represent data. The model considered here addresses both preattentional and attentional hearing as well as multisensory affects upon listening. Neurophysiological study of auditory attention identifies mechanisms underlying interactive listening experiences, including attentive and pre-attentive processing, and top-down vs. bottom-up interplay of attentional mechanisms (Fritz et al., 2007). Study of neuronal signals indicates that auditory processing is influenced by conscious focus of attention and expectation (Brechmann and Scheich, 2005; Voisin et al., 2006; Sussman et al., 2007). Expectation is a temporal process that may be largely supramodal (Nagarajan et al., 1998; Ivry and Spencer, 2004), meaning attention is mutually reinforced across vision, sound and touch. Multisensory context enhances a listener's attention to and discernment of sounds (Pastor et al., 2006; Best et al., 2007) including visual modulation of the audio cortex (Kayser et al., 2007). Teng et al. (2016) and Holcombe (2009) that visual cognition seems to have a timescale similar to audio for dynamic event perception. A listener's attention and expectation can influence physiological changes in the brain's plasticity of neuronal dispositions and responsiveness (Hillyard et al., 1973; Woldorff and Hillyard, 1991; Fritz et al., 2005). The resulting modifications in neural signal processing improve temporal performance and acuity of conscious recognition and identification of sounds (Spitzer et al., 1988; Cusack et al., 2004; Alain et al., 2007). Fritz et al. (2005) describes MMN, a supramodal neural mechanism for “oddball sensing” that detects unusual changes in surroundings. MMN indicates a listener's capacity for maintaining an heuristic focus on the transition from pre-attentional to new or recognized sounds.

It is important recall that Sonification does not interpret itself; it requires informed skill and learning how to listen. Heuristic Listening is introduced as an informal model describing multisensory cycles of action and observation that contribute to a listener's attentive process. Heuristic Listening defined here as *a clinically informed skill of multisensory enhanced attention to sounds that may be meaningful in a context of exploration and structured inquiry*. This skilled listening practice is similar to heightened everyday situations where a listener has an expectation a sound will occur but is uncertain of when the sound may occur. Heuristic Listening involves a listener's affective presence in an environment, with context awareness, attention, prediction, possible responses to false cues, and a response performed when an awaited sound occurs.

A model of Heuristic Listening requires representation of an on-going multi-temporal cognitive cycle, where sound events are disambiguated and articulated by the listener's actions, and where the listener's actions may also set expectations for sound events. Recognition of sounds and events occurs across a multisensory and supramodal cognitive cycle that continuously integrates multiple time layers, where multiple event recognition and observer actions overlap in multiple onsets and terminations. The reference example for this research is the LIDA model (Madl et al., 2011) describing a cognitive cycle comprised of Perception, Understanding, and Action Selection. LIDA identifies a 260–390 ms cognitive cycle of 200–280 ms unconscious processing, subdivided into Perception and Understanding, followed by 60–110 ms conscious Action Selection. These phases characterize the perception of audible attributes that occur within corresponding time windows, such as pitch, loudness, duration, and pulse.

#### Listening with multisensory interaction

Heuristic Listening describes enhanced time-sensitive expectation as a context for developing interactive sonification. Previously this author has studied listening as a function of physical interactions with emergent systems to generate sound, and introduced a kinaesthetic framework based upon multi-temporal cognitive cycles of multisensory attention and action (Choi, 2017b). Interactive sonification is designed to engage this dynamic temporal acuity as sound events are generated by a listener's actions. An action creates a time focus of attention that may elevate or supress neuronal responses depending on whether the sound is highly relevant or irrelevant to the conscious listening task (Martikainen et al., 2005). (Lange and Roder, 2006) reports that listeners who receive cues to aid prediction of audible event timing will experience temporally heightened neuronal attention. These findings suggest that a listener can elevate her level of attention to sonification by performing an active inquiry and having interaction with the experimental system being observed. Further, timing and intensity of a listener's exploratory actions will elevate expectations for corresponding changes in sounds. A listener's performance in terms of recognizing sounds may improve if the system provides multisensory attentional engagements. The present research provides three types of attentional engagements: visual cues from dynamic visualization of the experimental data, somatosensory cues from spatial orientation of physical movements within an interface (Hotting et al., 2003), and semantic cues representing users' actions in graphical user interfaces.

The neurophysiological basis of heuristic listening contextualizes a listener's experience of transition from expectation to recognition, reflecting the temporal dynamics of pre-attentive to attentive auditory cognition. A listener may be thought of as having a pre-attentional streaming segregation “buffer.” Incoming auditory signals accumulate in that buffer for durations that may be as much as several seconds. During the buffer period a listener's expectation can impact the rate of transition from pre-attentive to attentive state (Bregman, 1978; Molholm et al., 2005). A transition from pre-attention to attention is a moment of critical phase transition; it indicates the listener either recognizing a familiar sound pattern or learning an unexpected sound pattern. This phase transition is the attentive focus of heuristic listening; sounds may be unfamiliar and still be recognized to represent states of an underlying order. Identifying emergent properties in sounds engages heuristic listening in exploring regions of an experimental system by recognizing combinations of familiar and unfamiliar states in sounds. Unfamiliar sounds may be unstable or may exhibit unexpected transitions to new stable patterns. Listening memory plays a temporal role in uncertainty and recognition, exhibiting attributes of *ensemble coding* (Albrecht and Scholl, 2010), a mechanism of temporal statistical summary of information in a perceived scene. Ensemble coding is well-documented in visual summary of complex scenic features, and has been experimentally demonstrated in audible tone patterns (Piazza et al., 2013). The model of ensemble coding provides a foundation to account for a listener experiencing multi-temporal dynamic layers of sounds unfolding in time. In this example a listener simultaneously reflects on sounds previously heard, acquires sounds newly heard, and anticipates sounds yet to be heard (Ulanovsky et al., 2004). A moment of heuristic listening collocates the anticipatory, immediate, and predictive neurological processes of listening. Finally, heuristic listening implies a skill requirement supported by everyday listening experience, and a listener's capacity to become more acute by training and performing multisensory observation. These models are considered in design of the research methods applied in this work. Appendix 1 in Supplementary Material presents clinical examples of heuristic listening.

## Research methods applied to interactive sonification of emergent behavior

The goal of this research is to assess extensibility of a model as an interactive sonification framework, and to demonstrate its application for emergent behaviors. To perform assessment three methods are combined: *listening scenario classification, attunement*, and *control classification using sonification design patterns*. The combined methods are applied to sonify the Chua's circuit and Swarm Chemistry, and the research compares each application to show how these methods work together. The study aims to demonstrate the extensibility of a framework for interactive sonification by comparing variation and consistency in each method across multiple applications.

### Listening scenario classification

A sonification application includes a context whereby a listener acquires sounds in relation to other modalities of observation. This research introduces a sonification listening scenario as *a system design that prepares and enables listeners expectations of how sound generation is coupled to an experimental system*. Coupling requires that transformations of sounds correspond to salient emergent properties or state changes of the experimental system. A properly engineered sonification listening scenario yields an interactive learning pathway for listening skill acquisition, supported by a listener's understanding of the experimental apparatus.

A listening scenario is a construct designed with multimodal attributes that become part of a local listening environment; the listening experience is not determined solely by the audible sonfication output. Sounds are perceived in a highly subjective environment often fused in multisensory percepts. (Bregman, 1990) describes an *auditory scene* as a temporal superposition of “component” sounds comprised of multiple sources, some of which are not controllable, even in an isolated listening environment such headphones or an anechoic chamber. Environmental conditions generate component sounds that are attended at different levels of awareness. Neurophysiological pre-attentive mechanisms for audio stream segregation play an important role for differentiating and keeping track of multiple sounds from different sources in a complex auditory scene. Some but not all audible sounds rise to conscious awareness, a subset of audible sounds is noted as distinct events, a subset of these may draw a listener's attention. According to directed attention hypothesis (Welch and Warren, 1980; Andersen et al., 2004), multisensory percept plays a role in determining what sounds in the auditory scene are identified or disregarded based on what modality is dominant at any given moment. Both multisensory fusion and modality dominance can contribute to highly subjective listening. Supramodal auditory attention hypothesis states that stimulus driven shifts of auditory attention are controlled by a supramodal mechanism (Ward, 1994). A sonification listening scenario enabled with a multimodal interface engages multiple senses to inform the interpretation of sounds.

The auscultation training examples surveyed in Appendix 1 in Supplementary Material demonstrate how sounds representing known experimental states may be learned through observation of established cases. Classification of a sonification listening scenario can be formalized as a guiding template with examples of multimodal system norms and corresponding sound qualities. Sounds that are talismans of unfamiliar states or properties may thereafter be established through empirical observation. Deviance detection is highly sensitive in auditory perception (Fritz et al., 2007) and is coupled to neural responses in other sensory regions (Downar et al., 2000; Huang et al., 2005). Once a listener learns to recognize sounds that are a norm, exceptional sound events can be recognized.

In this research the classification of listening scenario is organized by classifying the affordances of the sonification system design: (1) type of affordance, (2) means to realize the affordance, and (3) indicative listener experiences related to the affordance. Table 1 presents a classification using on six types of affordance:

- audible quality,
- user interface,
- temporality,
- multisensory qualities,
- complexification, and
- learning provision.

**Table 1:** Classification of sonification listening scenarios organized by type of affordance.

**Affordance and means**	**Indicative classes of user experience**
**Affordance:** Sounds for a domain investigation **Means**: Sound generation and sound design	Identify sound qualities - what is heard Range of change exhibited by a sound The meaning of a sound in relation to experimental data; how a sound can be described with respect to the state of the simulation
2. **Affordance:** Interfaces for a domain investigation **Means:** Control paradigm	Sensory properties of a user interface including appearance, function, orientation, and ease of operation; Degrees of freedom provided in an interface Multidimensional control of an experimental system; the number of system control parameters accessed
3. **Affordance:** Perceptually compatible temporality of events **Means:** Scheduling of data	Synchronization or latency between the introduction of changes in control space, and the experimental system's response in phase space Synchronization or latency between phase space output and corresponding transformations of sounds Temporal resolution in a signal output from an experimental system and how this is reflected in audible temporal resolution
4. **Affordance:** Multisensory information **Means:** Engaging two or more modalities of an observer	Visual cues provided by an experimental control interface while sound is generated Visualization of data alongside sonification Enactive control of the domain investigation while listening; physical responses of interface
5. **Affordance:** Complexification **Means:** Generating and controlling levels of detail	Varying the ratio of experimental data to sound control data, with the purpose of modifying the level of detail of data mapped into sound Reduction of experimental data: Filtering the level of detail in data output from the domain investigation
6. **Affordance:** Learning Provision **Means:** Rehearsal for developing competence	Access to example scenarios of experimental states and associated sonification outputs Access to practice scenario interaction with rehearsal time to become acquainted with the domain investigation and sound generation

These attributes are device-agnostic and data source-agnostic. Examples of indicative user experiences provided in Table 1 represent potential criteria for qualitative and quantitative measurement. Appendix 2 in Supplementary Material presents a test case of the listening scenario classification method applied to independent published research involving personalized sonification of EEG feedback.

### Attunement of explorable space

Attunement is *an a priori process for conditioning a playable space for auditory display* (Choi, 2014a). The modeling of playable space is introduced from other applications of model-based interaction (Choi and Bargar, 2011; Choi, 2014b). Playable space is not a user interface; it is an enabling design for the development of interfaces. A model of a playable space may be formalized as a set of canonical relationships that enable the development of auditory interfaces for observing dynamical systems (Choi, 2014a). The concept of space as a working metaphor is common in scientific practices especially in applications of simulation and modeling^1^. The space metaphor is adopted to identify the formation of explorable regions of system states as having definable structure and function. For sonification applications the term *explorable space* is introduced to describe the collective high dimensional parameter space and circular causality of the extended sonification model illustrated in Figures 3, 4A–C. Appendix 3 in Supplementary Material summarizes functional explorable space comprising the extensible sonification model. The system is calibrated to ensure the sonification outputs and listeners' actions are meaningful with respect to the experimental system. Calibration involves adjustment of many control parameters, within each subsystem and between subsystems at each transfer function TF_1−3_. Attunement is a calibration method that systematically reduces the dimensionality of the parameter space in two stages, first stabilizing the subsystems' internal parameter values then adjusting the transfer functions. TF_1−3_ are referred to as *tuning functions* when the design of the transfer functions formalizes the attunement of the sonification framework.

To perform attunement, the range of parameter values applied at one TF may require adjustments at other TFs. Each TF requires tuning to optimize for isochronous differences between subsystems, adjusting oversampling and undersampling between subsystems to minimize artifacts generated by aliasing.

**Figure 5:**
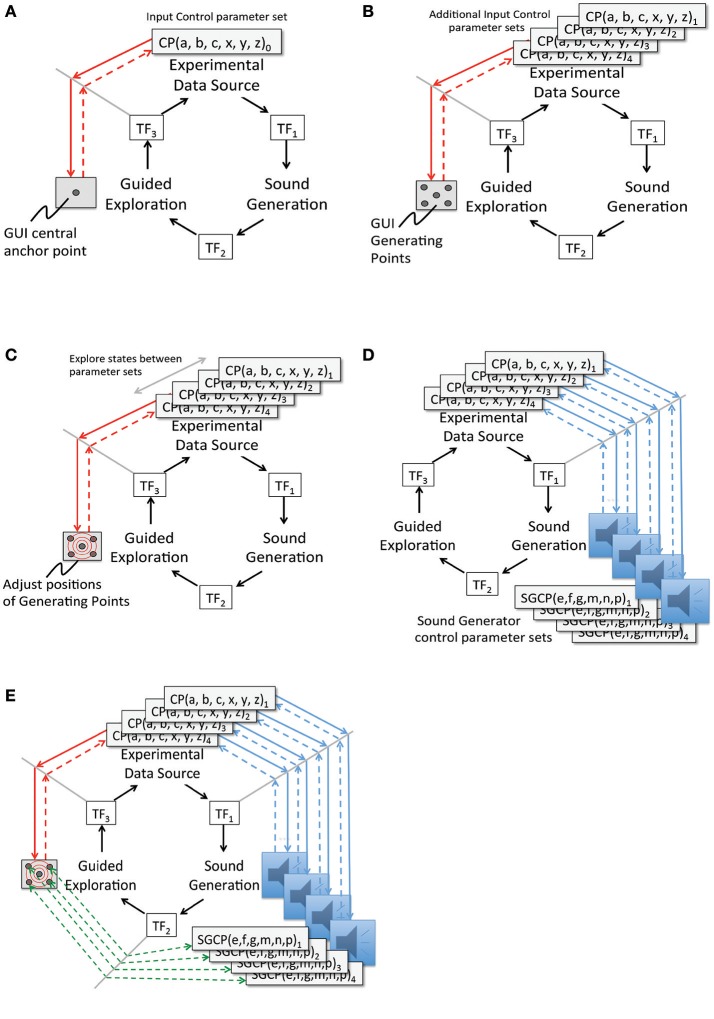
**(A)** Attunement Step 1: Between two subsystems, Guided Exploration and Experimental Data Source, define an anchor point in the interface to control the simulation. Process:(a) For Experimental Data Source: Initialize control parameter values of a known stable state. (b) For Guided Exploration: Select a central position in the interface and assign the control parameter values from Step A. (c) For TF_3:_ Encode the position assigned in Step B as the initial anchor point for attunement. (This is a multidimensional mapping from the set of control values A to the interface state B). Requirement: The initial mapping and interface anchor position are selected by human inference based on prior experience with the subsystems. **(B)** Attunement Step 2: Define *generating points* connecting the Experimental Simulation and the Guided Exploration interface. Process: (a) For Experimental Data Source: Identify control parameter sets of further stable simulation states. (b) For Guided Exploration: Select a set of new interface positions to correspond to each set of simulation state control parameter values from Step E. (Each set of control values E_n_ requires a multidimensional mapping to an interface position F_n_). For TF_3:_ Encode each interface position assigned in Step B as a generating point, GP_n_. Requirement: Interface positions for stable states are readily accessible and evenly positioned in the control interface. **(C)** Attunement Step 3: Establish a Scale of Transformation between generating points. Process: (a) For Guided Exploration: Explore interface positions between the Generating Points. (b) For Experimental Data Source: Observe changes in simulation Phase Space induced by intermediate positions in the exploration interface. (c) For TF_3_: Modify GP interface positions or GP control values to optimize symmetry of the interface, in terms of state transitions actuated when the interface moves between GPs. Requirement: Relative degree of change in the interface should correspond to relative degree of change in the simulation phase space. **(D)** Attunement Step 4: Between two subsystems, Experimental Data Source and Sound Generation. Process: (a) For Experimental Data Source: For each previously selected control state, observe characteristics of the data output. (b) For Sound Generation: Working with a selection of procedurally generated sounds, identify a set of sound control parameter values corresponding to each Data Source control state. (c) For TF_1:_ For each Data Source control state, encode a mapping from the experimental data output to a set of sound control parameters. (d) Requirement: Initial sounds are selected based on knowledge of sound design and previous experience with the dynamic qualities of the experimental data output. **(E)** Attunement Step 5: Across three subsystems, from Guided Exploration to Sound Generation. Process: (a) For Guided Exploration interface: Select each GP then select interface positions between generating points. (b) For Experimental Data Source: At each GP verify the state of the simulation phase space. Between GPs observe phase space transitions. (c) For Sound Generation: At each GP verify audible output. For interface positions between generating points verify audible transformations. (d) For TF_1_: Modify sound control space mappings to optimize for audible transformations that have a range of discernable differences corresponding to the dynamic range of the experimental data output. Requirement: Relative degree of audible change in sonfication should correspond to relative degree of change in the interface—implying a relative degree of change in the simulation phase space. Establishing normalized degrees of interface action and audible response, differences in audible transformations will indicate non-linear properties of the Experimental Data Source rather than artifacts of interface or sound control.

Figures 5A–E describe the process of implementing attunement. The process begins with the user interface by defining a set of discrete interface states as *generating points* (GPs). In Figure 5B the interface GPs are assigned to a selected set of control states. To simplify the process of mapping M-dimensional GPs to N-dimensional data control parameters, a manifold interface technique is introduced (Choi, 2000a). In Figure 5C the explorable interface space is calibrated with control regions of the Experimental Data Source. In Figure 5D sound control parameter data sets that determine audible features are aligned with selected states and features in the experimental data. In Figure 5E the listener associates audible features with generating points at the user interface, and audible transformations with explorable regions in the user interface. The attunement process is applied in cycles of iterative refinement while auditioning control input and sound output. Regions of interest in the experimental data are brought into correspondence with sound control data and audible transformations. Discovery of regions of interest in experimental data space may require iterative refinement. Adjustments to parameter values are applied at tuning functions TF_1_ and TF_3_. Tuning function TF_2_ is a representation of a listener's performance of heuristic listening.

### Control classification using sonification design patterns

A sonification design pattern (SDP) is a control structure for generating a data-driven audio stream. SDPs selectively control audible features to optimize audibility of features exhibited in experimental data. SDP structure is agnostic to audio content in the sense that one structure may control many types of sound generators. SDP are informed by Alexander's concept of design patterns (Alexander et al., 1977) developed for architecture, and SDP may be considered members of the superset of design patterns used in software engineering (Gamma et al., 1994), as SDP define procedural audio using instruction sets for sound generators. This author introduced the SDP method for interactive sound generation using non-linear simulation data (Choi and Bargar, 2014), and has applied SDP in multimodal performance with evolutionary systems (Choi, 2017a). SDP facilitate attunement by classifying control data features to align with audible features. In the sonification framework SDP are located in the Sound Generation subsystem and receive data from the output of TF_1_. SDP control parameters define the control space of the Sound Generation subsystem (Figure 4B), where SDP functions are modulated by the experimental data from TF_1_. SDP may be designed to control many different sound palettes. In the Swarm Chemistry case studies two SDP examples are applied.

Duration is the first organizing attribute for all SDP. Control parameters are defined for SDP depending upon the duration range and the type of sound generator to be controlled, presented in Table 2. Duration range of an SDP refers to signal processing time required to generate an audible signal combined with perceptual time required for a listener to register the audible attribute or pattern. We identify five sets of SDP attributes with characteristic duration ranges: SDP 1 controls pitch change and loudness change; SDP 2 controls timbre, resonance and filtering; SDP 3 controls sound source location and spatial cues; SDP 4 controls distinct sound events; and SDP 5 controls patterns made up of multiple sound events. A sound event must have sufficient duration to be audibly reproducible—meaning a pattern is recognizable because of its organization of elements (see Wiener in section Introduction: What Do We Listen to When We Listen to Data?). Up to a limit, a pattern is identifiable independent of tempo (rate of change), frequency range, and other attributes. Table 2 provides duration ranges of five SDP classes. Appendix 4 in Supplementary Material reviews auditory perception at multiple time scales, relevant to SDP duration classification and the audibility of data.

**Table 2:** Sonification design pattern classification by duration.

**SDP timescale, micro to macro**	**Audible attributes that generate features in SDP**	**Duration range of audible SDP attributes**
SDP1—micro	Pitch change, loudness change	50–200 ms
SDP2—micro	Timbre, Resonance, Filtering	200–500 ms
SDP3—meso	Sound Source Location and Distance cues; Spatial cues	450 ms–2.0 s
SDP4—meso	Sound Event (from onset to evolution to termination)	500 ms up to seconds
SDP5—macro	Patterns of Rhythm, Tempo, Spoken Words, Melody	1 s up to seconds

*Multiple SDPs are combined to develop an audible palette. Audible features of SDP structure are dependent on the sound design and attributes of the sound generator. Features in SDP classes can be controlled independently at a sound generator while the audio output may elicit auditory co-dependencies due to psychoacoustics of perception*.

## Results: applying the proposed sonification framework to multiple experimental systems

An extensible model of interactive sonification is introduced in section Data Acquisition Model for Sonification Signal Processing as a candidate sonification framework. The extensibility of the framework is compared across three sonification case studies using two experimental simulations that generate emergent behaviors. One case is implemented for Chua's Circuit and two for Swarm Chemistry. The three cases are each presented in terms of attunement method at TF_1_, listening scenario design, and observed outcomes with interpretation. Each implementation adopts a different level of abstraction between the experimental data source and the sound generation. The collective outcomes compare the framework performance in representing the experimental implementations. Performance measures include:

- Accuracy and relevance of the framework representation of an experimental sonification implementation
  ◦ Accuracy of framework representation of an experimental system in terms of functional components, their sequence and their relationships
  ◦ Whether the framework lacks components required in the experimental system, or the framework includes components extraneous to the experimental system
- Using the framework to compare multiple experimental sonification implementation
  ◦ Identify model components that have common roles in two+ implementations
  ◦ Identify model components that have different roles in two+ implementations
  ◦ Identify experimental components that have inconsistent relationships with model components in two+ implementations
- Using the framework to compare solutions for emergent behavior adopted in multiple sonification implementations
  ◦ Similarity and difference of challenges presented by different types of emergent behavior
  ◦ Accuracy of the solution space representation in the framework, compared to the application of the solution in the experimental system.

### Sonification framework applied to Chua's circuit simulation

**Figure 6:**
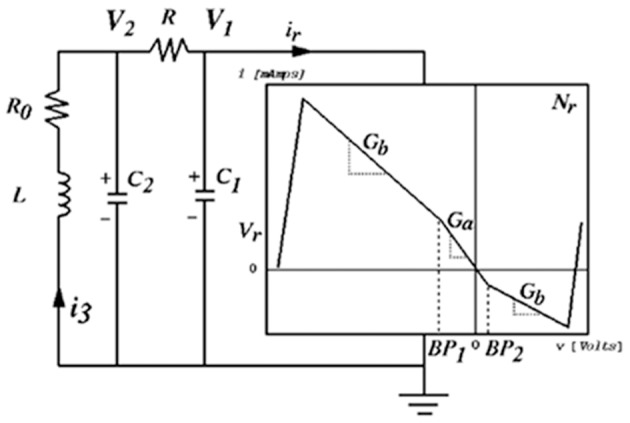
Chua's circuit is comprised of seven elements: one locally active resistor where energy is introduced, three energy storage elements—two capacitors and a resistor, and one resistor programmed with a piecewise non-linear transfer function that induces chaotic behavior.

**Figure 7:**
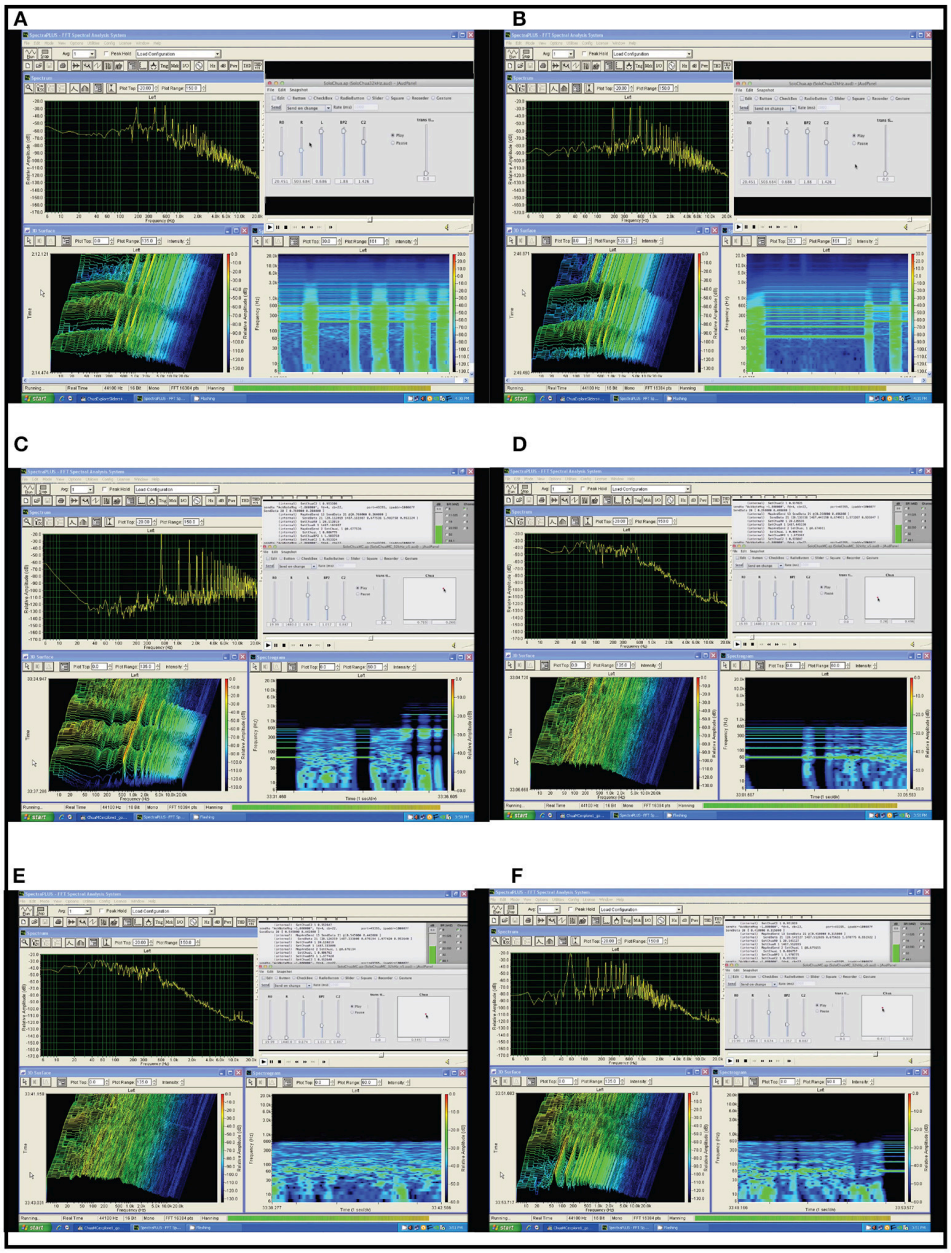
Six examples of emergent behavior in Chua's circuit oscillation. Each example presents, in the upper left a frequency domain energy distribution (spectral envelope) of the current time step; in the lower left a frequency domain waterfall time series with amplitude heat map; in the lower right a 6 s time series of frequency domain with amplitude heat map, the most recent time step at far right; and at upper right the user's graphical interface for voltage control of 5 of the 7 circuit components, showing the current values of control voltages. **(A)** Quasi-periodic attractor exhibiting intermittency. A periodic attractor producing a harmonic tone with fundamental frequency near 200 Hz and additional periods producing additional harmonic tones at integer multiples above the fundamental. The attractor exhibits intermittent bursts of chaotic behavior creating an irregular rhythm of noise bursts interrupting the tone. Intermittency emerges in phase space at the boundary of a stable attractor region and an unstable chaotic region. Voltage control change is applied serially to one circuit component at a time using individual linear potentiometers. **(B)** Rapid transition from one quasi-periodic attraction to another. At the start of the time series a periodic attractor exhibiting intermittent bursts of chaos produces a harmonic tone with fundamental frequency near 80 Hz and additional periods producing harmonic tones at integer multiples above the fundamental. Around 5 s in the time series a change of control parameter shifts the oscillation to a different periodic basin of attraction producing a harmonic tone with fundamental frequency near 200 Hz and additional harmonic tones at integer multiples above the fundamental. Note the third highest period in the original attractor becomes the fundamental period in the second attractor. Voltage control change is applied serially to one circuit component at a time using individual linear potentiometers. **(C)** Rapid transition to Upper Limit Cycle attractor. At the start of the time series a periodic attractor exhibiting intermittent bursts of chaos produces a harmonic tone with fundamental frequency at 60 Hz and additional periods producing harmonic tones at integer multiples above the fundamental. Around 5 s in the time series a change of control parameter shifts the oscillation to an upper limit cycle attractor, which is nearly periodic at 500 Hz producing a fundamental harmonic tone and an ascending series of tones at integer multiples of 500 Hz. Note in the lower two windows during the bursts of chaos the amplitude heat maps show the frequency spectrum energy remains concentrated around the periods of the neighboring attractor. Voltage control change is applied in parallel to five circuit components using the cursor in the plane on the right side of the GUI. This is the 2D control surface for the manifold interface, mapping each 2D position to a 5D control signal. **(D)** Onset of Chaos. At the start of the time series a stable periodic attractor produces a harmonic tone with fundamental frequency at 60 Hz with additional periods producing harmonic tones at integer multiples above the fundamental. By introducing voltage control changes the system moves gradually out of the basin of attraction and falls into a chaotic region. Note in the lower two windows during the onset of chaos the amplitude heat maps show the frequency spectrum energy remains concentrated around the periods of the nearby attractor. Voltage control change is applied in parallel to five circuit components using the cursor in the plane on the right side of the GUI. This is the 2D control surface for the manifold interface, mapping each 2D position to a 5D control signal. **(E)** Chaotic Oscillation. A chaotic attractor produces oscillations constituting many rapid transitions between multiple periodic regions. The result is a noise-like signal with a faintly audible tone center shifting across the frequency range between 50 and 100 Hz. This persistent weak attractor region is visible in the spectral envelope of the upper left image and in the amplitude heat maps of the two lower images. Voltage control change is applied in parallel to five circuit components using the cursor in the plane on the right side of the GUI. This is the 2D control surface for the manifold interface, mapping each 2D position to a 5D control signal. **(F)** From Chaotic to Periodic Oscillation. At the start of the time series a chaotic attractor produces oscillations constituting many rapid transitions between multiple periodic regions. The result is a noise-like signal with a faintly audible tone center shifting across the frequency range between 50 and 100 Hz. This persistent weak attractor region is visible in the amplitude heat maps of the two lower images. During the time series example control voltage change is applied steadily and the oscillation exhibits intermittent stability then becomes stable on a periodic attractor at 50 Hz, with higher periods at integer ratios of the fundamental. Voltage control change is applied in parallel to five circuit components using the cursor in the plane on the right side of the GUI. This is the 2D control surface for the manifold interface, mapping each 2D position to a 5D control signal.

Chua's circuit is an experimental paradigm that satisfies the working definition of a chaotic system. It is well-suited to observe and analyze emerging behavior in physical systems, being comprised of the minimum number of elements required for a circuit to demonstrate chaotic behavior (Kennedy, 1993), illustrated in Figure 6. Chua's circuit has been implemented as a physical device and as a digital simulation using numerical models of the circuit elements^2^. This author has applied interactive sonification to both physical and digital implementations (Choi, 1994). Many classes of biosignals exhibit oscillations with functional chaotic properties. Simulations are used to study and classify these behaviors, and Chua's circuit exhibits a diverse repertoire of intermittency, quasi-periodic patterns, and chaotic signals. (Kozma and Freeman, 2017) describe intermittent series of synchronized metastable brain states as essential to neocortex processing, with interimittency enabling serial phase transitions that advance cognition from one metastable pattern to the next, a model known as the cinematic theory of cognition. Haken (1983) associates intermittent synchronization with information transfer between levels of neurons. (Tsuda et al., 2016) identifies biological dynamics under constraints such as embryonic development and differentiation of cortical functions, as having dependencies on properties of non-equilibrium systems such as bifurcation and attractor formations such as those well-observed in Chua's circuit, and outlines a critical connection from chaotic dynamics to the capacity for macroscopic self-organization in biological systems, providing mathematical models. Although the Chua's circuit is a deterministic system, emergent oscillatory features cannot be predicted by the states of the individual Chua oscillator elements or from preceding states of the output signal. Figure 7 presents examples of emergent behaviors of the Chua's circuit oscillation. In addition Chua's circuit exhibits *hysteresis* (Zhong and Ayrom, 1985) where a given control state may generate more than one oscillatory state, resulting in more than one set of audible features for a set of control values. The sequence and duration of control state changes and the corresponding sequence of oscillation states influence hysteresis. This emergent behavior presents significant challenges to generating reliable sonification. Appendix 5 in Supplementary Material presents a technique adapting attunement for sonification to compensate for hysteresis.

#### Case study 1: TF_1_ attunement for Chua's circuit as a sound generator

**Figure 8:**
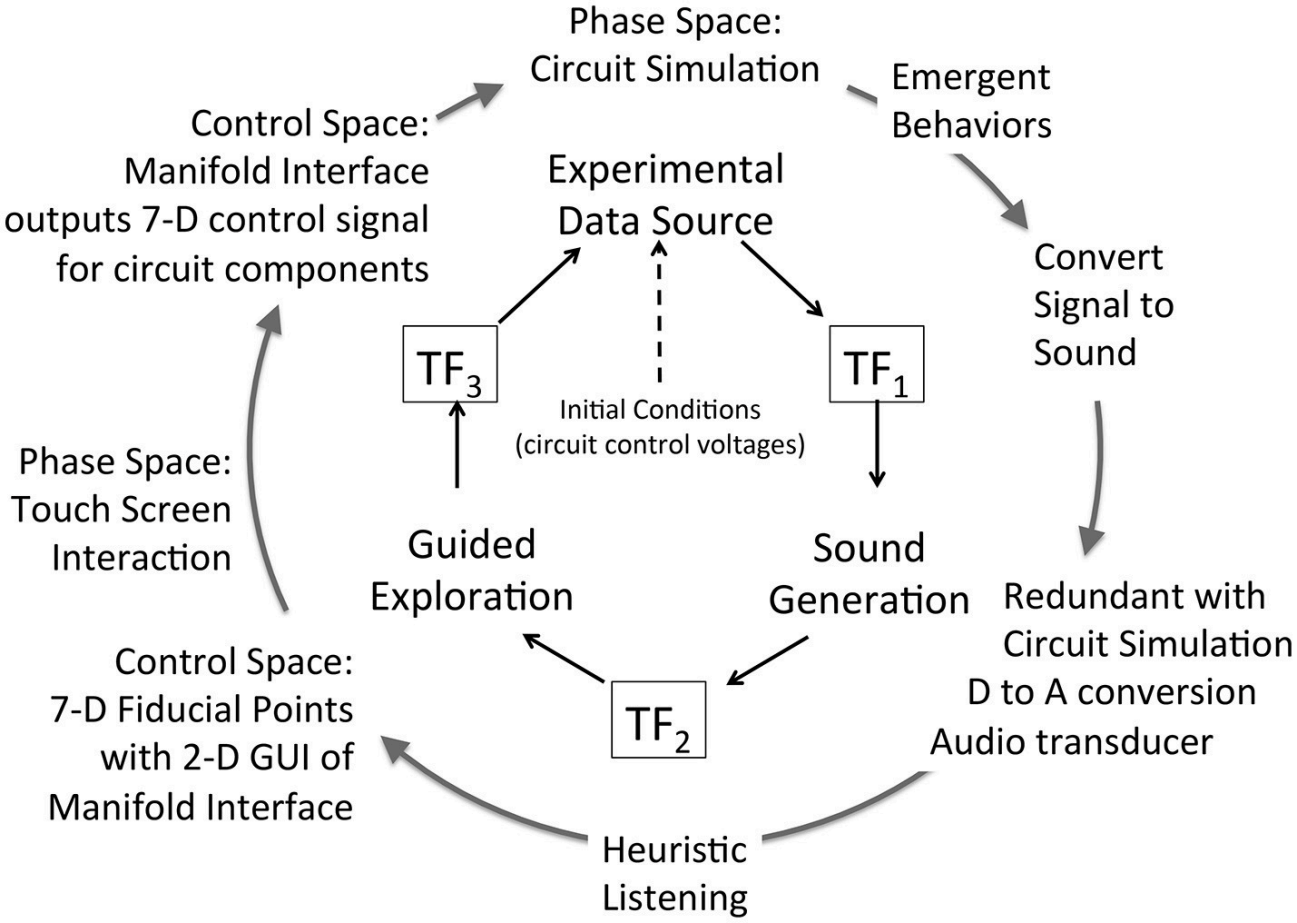
Attunement applied to Chua's circuit using the sonification framework. The Experimental Data Source is Chua's circuit controlled by voltages of seven electronic components. The circuit oscillation range is tuned to a human-audible frequency range. The Sound Generation system is redundant with the circuit simulation; the circuit oscillaton is sampled at a capacitor and converted into an audible signal. The listener acquires the audio signal while engaging a 2D interface, which visualizes fiducial points of stable control regions and enables exploration of other control regions. The Manifold Interface enables the 2D GUI to represent a 7D control space, continuously and differentially in a manifold subset of control space. Listener actions at the interface are converted to a 7D control signal and applied to the control voltages of circuit components. This enables heuristic listening in response to changes induced in the phase space of the circuit oscillation. **Table 4** details the functional components in this figure.

The signal output by Chua's circuit oscillation exhibits properties that may be human audible. The attunement is illustrated in Figure 8 and **Table 4** details functional components. The circuit oscillation is located in the phase space of the Experimental Data Source, with a seven-dimensional control space. Sonification is implemented by enabling the listener to access an audible signal from a direct data stream of the circuit's state.

Attunement at TF_1_: The tuning function TF_1_ applies scaling to the control voltages of circuit elements to establish oscillation in a human-audible frequency range. TF_1_ also identifies a single circuit element, capacitor C2 (Figure 6), where the oscillation signal is extracted and routed to digital-to-analog conversion to generate an audible signal.

Listening Scenario: Listening directly to the experimental data in real time creates an affordance for highly interactive control of Chua's circuit, requiring covariance of seven circuit elements for agile navigation of non-linear phase space. A manifold interface technique was developed to facilitate interactive covariance for *n*-dimensional control parameters (Choi, 2000a). To regulate sound generation from emergent behaviors, *fiducial points* are a type of generating point used for attunement (Figure 12). Appendix 5 in Supplementary Material introduces the manifold interface technique for structured high dimensional control and discusses the use of fiducial points to empirically tune the exploration interface.

Observed Performance: The manifold interface tuned with fiducial points enables a listener to control the Chua's circuit in real time. Using the interface to covary seven control parameters, a listener can develop agility to manually guide the circuit states through regions of interest. Guidance from audible features improves the precision of navigating control regions that generate emergent behavior. Emergent behaviors are often exhibited in unstable regions that border unresponsive regions such as fixed points—where oscillation ceases, and limit cycles—where oscillation is fixed in a sine wave. Attunement aids exploration by identifying fiducial points with audible characteristics that indicate when the circuit is approaching undesirable regions. By attentive listening and applying micro-modifications to control parameters the listener can navigate circuit states away from a transition region or nudge the circuit state from one attractor region to another. This approach makes use of the sensitivity of unstable regions, and very small changes introduced at the manifold interface will influence the signal trajectory at boundaries of phase transitions.

Interpretation: Fiducial points can be used to mark behavioral trends in control space regions, and these points used to guide listeners through unstable regions in phase space. An interface providing stable control points bordering unstable regions supports the practice of Heuristic Listening by enhancing the reliability of free navigation in complex control space. These methods are compatible with the use of signal analysis and modeling to identify points in phase space that represent, predict and influence behavioral trends in chaotic systems. (Schiff et al., 1994) report a chaos control technique that applies signal analysis to identify unstable fixed points for learning the directions of signal approach and divergence. A small perturbation in the signal is introduced in these regions to prompt the signal to adopt preferred pathways. Faure and Korn (2001) report the use of recurrences plots in regions of phase space to characterize signal tendencies in the region and to predict the evolution of dynamics in the region, to a proximate future. These regions are sites for influencing phase transitions and signal behavior by applying weak perturbations to the control space. Attentive listeners using a manifold interface can explore highly unstable boundary conditions and influence the circuit to maintain quasi-stable states.

### Sonification framework applied to Swarm Chemistry simulation

**Figure 9:**
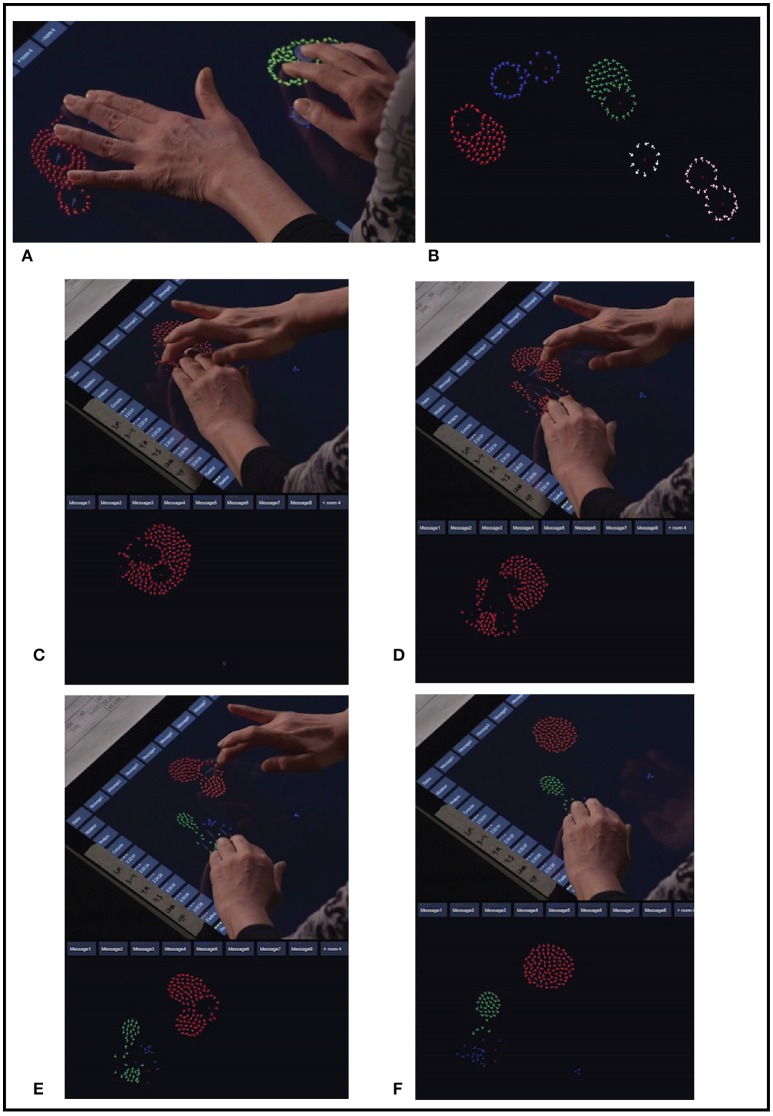
Two examples of Swarm Chemistry emergent behavior induced by an observer's interaction. Swarm agents are visualized on a touch screen interface. Each touch point generates a SuperAgent in the swarm simulation control space. Agents' autonomous responses are determined by their behavioral rules for social interaction. Feature recognition is applied external to the simulation and is used to visualize agent clusters using color. **(A,B)** Two views of swarm agents gathered around 10 touch points from observer's interaction. In **(B)** five clusters are recognized by feature detection and visualized using color. Swarm clusters are an emergent property formed by agents' collective behavior. Individual agents are not aware of their membership in clusters. **(C–F)** A sequence illustrating induced bifurcation of one agent cluster into two clusters. In **(C,D)** an observer applies touch to agents in one cluster and leads the agents in two separate directions. In **(E)** some agents' collective movements break away from the original cluster and form a second cluster. Feature detection is applied external to the simulation and the two clusters are visualized using two colors. In **(F)** the observer has removed the touch points and the two clusters exhibit symmetry through the agents' social interaction.

Swarm Chemistry (Sayama, 2012) is based on Reynold's “boids” system (Reynolds, 1987). Sayama implements heterogeneous agents, each agent having autonomous social tendencies expressed as movement, and awareness of other agents sharing the movement space with social tendencies. The simulation specifies 100 to 300 agents, each agent initialized with movement tendencies, perceptual radius, social responsiveness and a random initial position and velocity. Each agent is visualized as a low-polygon 2D graphical object animated in a bounded plane. An agent moves autonomously with a defined probability of random velocity until another agent enters its perceptual radius. At each time step in the simulation every agent responds to all other agents that are within its perceptual field. Agents' movement responses express these parameterized tendencies: straying, cohesion, alignment, separation, whim, and pace keeping, presented in Table 3. An agent's tendencies are quantified as strength of attraction to the average position and average velocity of all perceived neighbors, an imperative to avoid collision, a probability to move randomly, and strength of tendency to approximate its own average normal speed. Parameters for these attributes for each agent define the control space of the simulation. The state of an agent includes its attribute values, its current position in the movement plane, and its movement history required to determine acceleration and probability. At each simulation time step the movement response of each agent is solved for its attributes with respect to all other perceived agents, and the resulting positions of all agents are collectively updated. Other than state required for these calculations an agent has no memory of prior movement or location, and agents have no top-down spatial view of the movement area or of other agents' formations or positions. A system was implemented for human socialization with agents enabling touch screen interventions in the simulation (Choi and Bargar, 2013). With or without external intervention collective patterns emerge across groups of agents. Figure 9 illustrates examples of swarm agents' emergent behavior, induced by an observer using an interface to interact with agents' social tendencies.

**Table 3:** Swarm Chemistry behavior control parameters and social conditions.

**Agent control parameter**	**Agent's behavioral tendency**	**Social conditions**	
		**Alone**	**With others**
Straying	Move randomly if no other agents are in perceptual range	Required	
Pace Keeping	Approximate current speed to normal speed	Optional	Optional
Cohesion	Move toward the average position of other agents in perceptual range		Required
Alignment	Accelerate toward the average velocity of other agents in perceptual range		Required
Separation	Avoid collisions with other agents		Required
Whim	Move randomly with a given probability		Required

#### Procedural sound generation for Swarm Chemistry sonification

**Figure 10:**
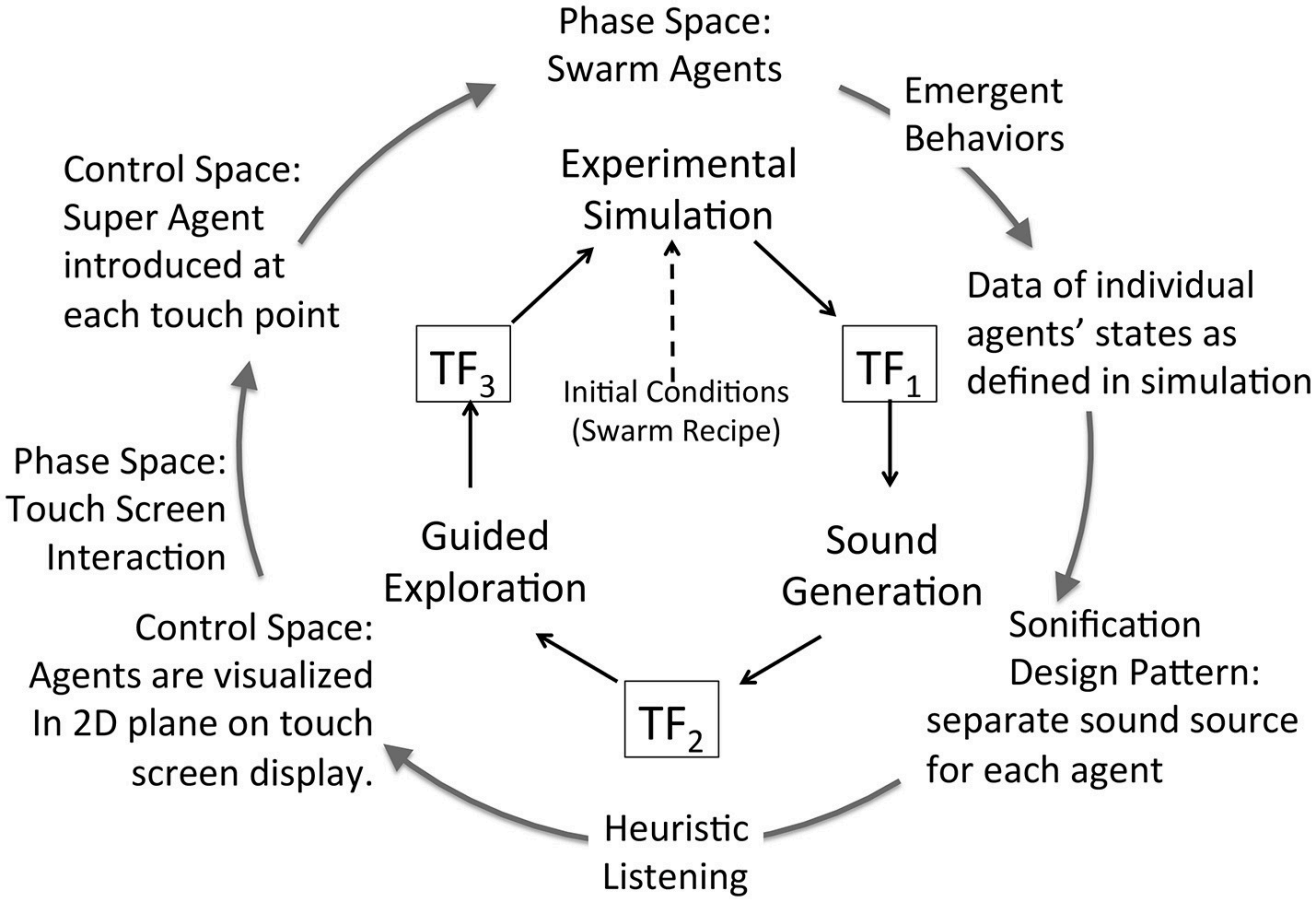
Attunement applied to swarm agents' simulation using a “literal” method, generating a separate sound for each agent, and mixing sounds in aggregate to render emergent patterns from agent data into auditory information. The simulation defines agents in 6 dimensions (Table 3) and several dimensions are selected from these data streams to provide control data for sound production for each agent. The sound is rendered as an aggregate mix for *n* agents; in the reported research *n* = 200. The listener acquires the audio signal while engaging a 2D interface, which visualizes agents' movement in a 2D plane. The listener may touch the screen to influence agents' behavior. At each touch point a SuperAgent is introduced in the simulation control space and the agents respond to touch points as if they are regular agents. This enables the listener to apply social influence to modify agents' collective behavior. **Table 5** details the functional components in this figure.

**Figure 11:**
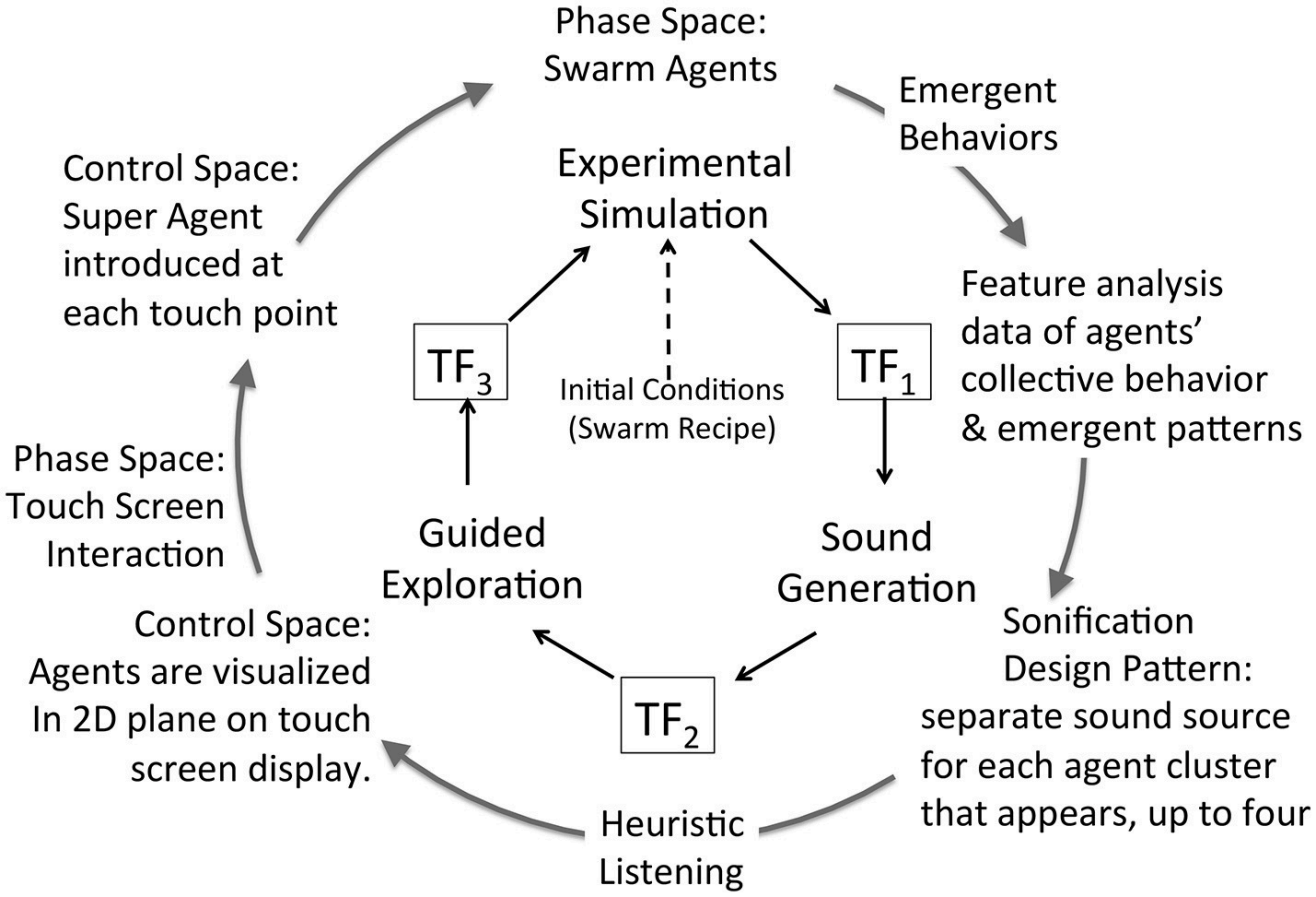
Attunement applied to swarm agents' simulation using a pattern recognition method. Cluster formation among subgroups of agents is the primary feature reported. Secondary statistical data about each cluster is also reported, such as average velocity. Several dimensions are selected from these data streams to provide control data for sound production. The four largest clusters are selected as the sources of control data, and an independent sound source is generated to sonify the data of each cluster, up to four clusters. The sounds are transformed in direct correspondence to feature data measured in each cluster. The listener acquires the audio signal while engaging a 2D interface, which visualizes agents' movement in a 2D plane. The listener may touch the screen to influence agents' behavior. At each touch point a SuperAgent is introduced in the simulation control space and the agents respond to touch points as if they are regular agents. This enables the listener to apply social influence to modify agents' collective behavior. **Table 5** details the functional components in this figure.

Swarm Chemistry simulation data cannot be converted directly into an audible signal. The Sound Generation subsystem for Swarm Chemistry adopts procedural sound synthesis, enabling a broad range of auditory representations including sonification design patterns (section Control Classification using Sonification Design Patterns). For two cases studies, two methods of sonification are implemented as alternative attunements of transfer function TF_1_ in the sonification framework, illustrated in Figures 10, 11. Case Study 2 (section Case 2: TF1 Attunement for Parallel Data Streams of Many Agents) applies individual agents' data to directly control an equivalent number of individual sound sources. This method relies upon multiple sound source aggregate interaction to generate emergent features analogous to visual pattern emergence. Case Study 3 (section Case 3: TF1 Attunement Using Feature Recognition Data) applies statistical measures to detect the emergence of salient features. This method applies swarm data indirectly via pattern recognition data to control sound synthesis. To maintain control for comparative assessment, Case studies 2 and 3 adopt a common attunement of transfer functions TF_2_ and TF_3_, including a common Exploration Interface for the listener to interact with the Swarm Chemistry simulation. Common attunement of TF_2_ and TF_3_ enables the comparison of two sonification methods at TF_1_ applied to a common source of emergent behaviors. At TF_2_ the Exploration Interface is implemented using a multi-touch surface to display a graphic visualization of swarm agents. The visualization becomes an interface by enabling the user to interact directly with swarm agents by touching the screen. Appendix 6 in Supplementary Material introduces the *super agent* mechanism that enables listeners' social interaction with agents in simulation phase space. Adopting swarm data visualization as an interactive interface requires sounds generated in real-time presented synchronously with the visualization. As an audible channel parallel to visual patterns, sonification may provide either redundant or complementary information. Dynamic patterns emerging from agents in aggregate pose a challenge for fidelity of data representation in sound. Emergent patterns are transient features and introduce uncertainty in rendering these features in sound. Patterns that emerge visually in the swarm data may not emerge in sound, depending upon the relationship between the swarm data and the sound generator. This difference is demonstrated in Case Studies 2 and 3.

**Figure 12:**
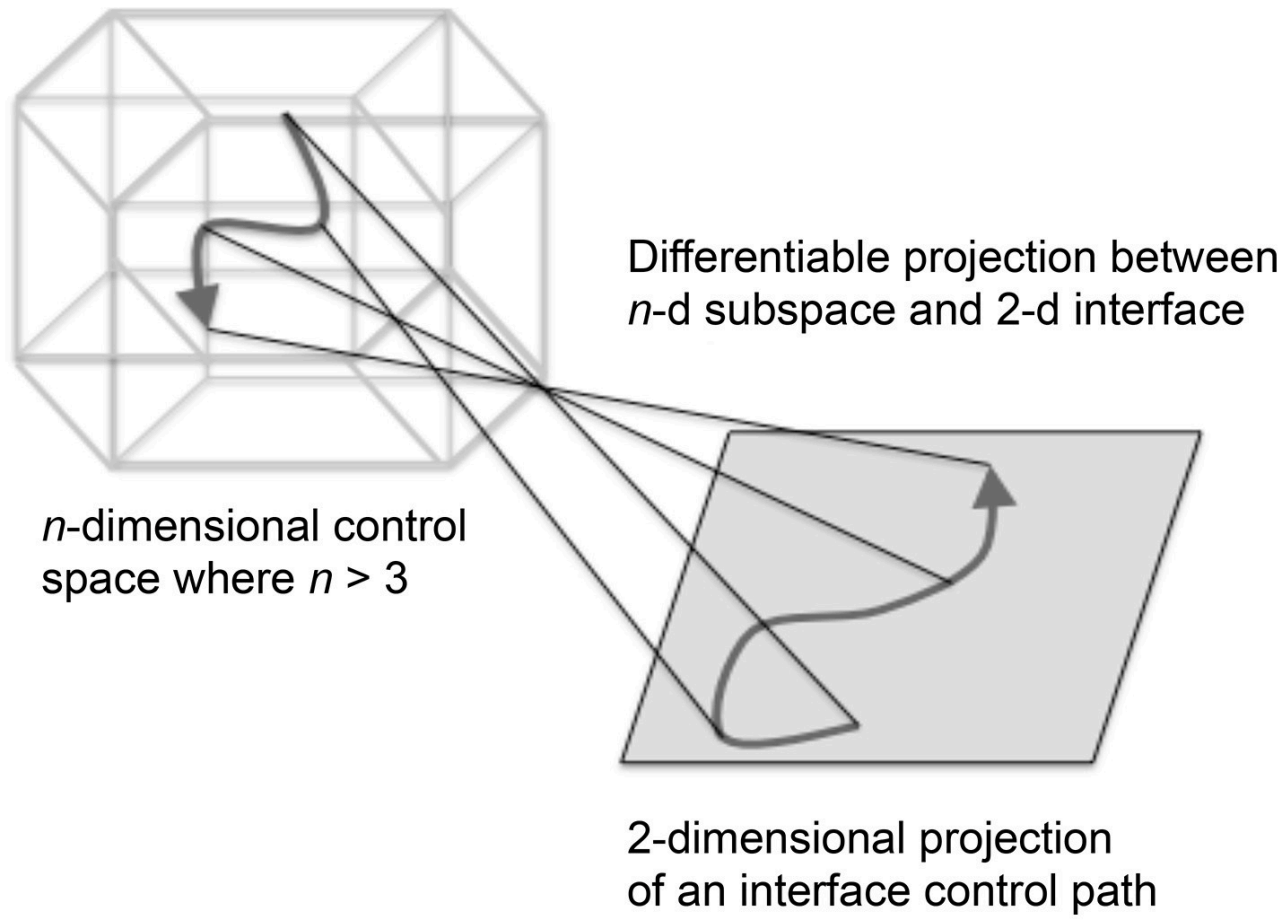
Schematic illustration of a manifold interface with a control path and four fiducial points. A manifold is a locally Euclidean topological space representing linear ranges of *n* control parameters in *n*-dimensional Euclidean space, where each point in the space is an *n*-tuple of real numbers corresponding to a unique set of control values of a parameterized system. An *n*-dimensional control path is illustrated on the left and the path on the right is generated by differentiable 2D projection from a bounded sub-region of *n*D space. The 2D actuation space is differentiable and bi-directional with the *n*D sub-region. Fiducial points are indicated by the endpoints of the four lines projected between spaces.

#### Case 2: TF_1_ attunement for parallel data streams of many agents

To sonify the data of individual agents the movement of each agent is measured and transmitted as control data for a corresponding sound source. Assigning a unique sound source for each agent may be referred to as a “literal” method: *n* agents generate *n* concurrent data streams applied to control *n* individual sound sources. For swarms where *n* = 300 to 500 this data is high density and the sound computation is intensive for real-time interaction with the swarms. Auditory perception tends to limit recognition of concurrent sound sources; the attunement of many parallel sound streams anticipates their collective mixture. To generate sound for each agent a harmonic tone with uniform frequency spectrum, frequency range, and duration were applied to each agent's data to normalize the audibility of all agents. A high-density sound mix was anticipated to mask sounds of individual agents so that collective attributes will emerge. This attunement is illustrated in Figure 10 and **Table 5** details functional components.

Attunement at TF_1_: To preserve a literal association to the visualization of agents, each (x,y) position was mapped to an auditory range that varied over fundamental frequency (pitch) and stereo position. To be highly literal in correspondence to the visualization, each agent's y-axis position was mapped a pitch (low to high with the position of the agent) and each x-axis position was mapped to stereo position (left to right with the position of the agent). These associations were selected as the most elementary with respect to simultaneous visual display of agent positions. Phase space dimensions are scaled to 1024 × 768 pixels; agent size is 4 × 4 pixels and agents move stepwise by units of 1 pixel.

Listening Scenario: The sonification design is hypothesized to render only general correspondences perceivable. For example, using a stereo field to represent lateral position of sound sources provides a range of perhaps a dozen virtual source positions that can be distinguished (Begault, 1994; Pedersen and Jorgensen, 2005), and then only when agents are in a relatively tight cluster. For 300 agents the tuning anticipated that when at least 70% (210 agents) are in a single cluster occupying no more than 15% of the x-axis range (153 pixels), a dominant stereo position will be audible. For pitch perception the frequency range of the y-axis was scaled between 800 and 1,200 Hz so that agents in close proximity will generate a focused pitch center (the harmonic ratio of 2:3 is a Perfect Fifth, a little more than half the perceptual difference of an octave). This tuning anticipates, when at least 60% (180 agents) are in a single cluster occupying no more than 15% of the y-axis range (114 pixels), that on the y-axis a dominant pitch center will be audible. This sonification design anticipates widely dispersed agents to render a broad tone cluster with no directional imaging of a sound source location. Evaluation aimed to test whether a separation of agents into two discrete clusters would be audible as two sound clusters separated by relative differences in pitch center and perceived source position.

Observed Performance: The literal attunement did not convey audible patterns that were easily recognized, compared to the clarity of visual patterns from the same data. Audible features were much less distinct than visible features of agent distributions and clusters. Source position and pitch center overall were weak. The dominant sound was a quiet broad pitch spectrum across the frequency range and stereo field. Even the most highly centralized clusters were weak in conveying pitch center and source position compared to the background sound. Stray agents in small percentage were sufficient to interfere with imaging. This lack of distinction is likely a result in part of the limited sound palette of the SDP. Many audible attributes were not included in data-driven transformation, and a higher-order sound pattern was not applied. The audible profile of the simple tones does not provide strong coherence for rendering spatial relationships among the agents.

Interpretation: The distributed sound source sonification is ineffective, in particular when compared to visualization features. Applying agent data to modulate multiple audio parameters would likely improve audible imaging of source location and pitch center. Interaction with the swarms improved recognition of audible features but the sound alone could not be used to perform accurate “blind” interpretations of swarm patterns.

#### Case 3: TF_1_ attunement using feature recognition data

As an alternative to direct sonification of each agent, sonification may use data generated by pattern recognition techniques applied to statistical analysis of agents' collective behavior. Cluster formation is a common emergent pattern, occurring when agents separate from a large swarm or gather from dispersed positions forming one or more cohesive subgroups. A swarm may self-organize into a variable number of clusters and undergo autonomous phase transitions where one cluster spontaneously separates into two, or two clusters come into proximity and merge into one. Symmetry and asymmetry of cluster shape is another common emergent pattern, with clusters achieving circular shapes at some times and other times dynamically deforming along the x- or y-axis. Change of density is another common emergent pattern, varying the number of agents in a cluster with respect to the cluster's geometric area (its visible “size”). This attunement is illustrated in Figure 11 and **Table 5** details functional components.

As swarm agents collectively generate patterns the individual agents' actions exhibit emerging aggregate social dynamics across subsets of agents. Sonification can reflect different levels of information, from individual agents' details to emergent collective patterns. Sonification design patterns used in Case Study 3 expose multiple levels of detail in sound production and enables design of a scalable relationship between level of detail in data and level of detail in sound transformation. Adjusting the relationship between data and SDP can modify level of detail that is transforming sound.

Attunement at TF_1_: A pattern recognition process is located in TF_1_ to detect and quantify swarm aggregate features (Figure 11). Target patterns are identified in advance from a selection of known emergent features. A recognition method is used to dynamically detect pattern formation in agents' positions. Data of all agents' positions is routed to the recognition process where the number and membership of clusters is determined at each time step. Each cluster is measured in area (visible size), density (number of agents/area), and symmetry or asymmetry (circular or deformed shape). The resulting pattern data is applied to control the SDP, which are designed with affordance for multiple clusters. Additional sonification is applied to phase transition events, for example signaling the separation, merging, and deformation of clusters.

Listening Scenario: The sonification design hypothesized that sound sources could be used to represent clusters and that listeners could understand audible features observed up to a limit of four concurrent sound sources, representing the largest four clusters. We further hypothesized that sound transformations can mirror visual transformations to enhance multimodal attention focus. SDP were designed in relation to the target feature set. For each target pattern, measurements used a single-value normalized scale [0,1] to quantify recognition, and this data controls the sound palette. Intermediate values between several target patterns generate corresponding interpolations of SDP related to each pattern.

Observed Performance: Applying feature detection data to control sonification design patterns, the audible transformations clearly corresponded to visible features that were measured by pattern recognition. In addition the sounds' qualitative differences enhanced the visualization by enabling fine-grained audible comparisons of relative size and dynamic properties of clusters. Listeners did not report confusion from four sound sources, in part because of coupling to visual cues. The range and variety of sounds enhanced quantitative understanding of the swarms without requiring a one-to-one relationship between the number of agents and the number of sounds. Sounds and their transformations were designed to represent the range and variety of features that comprise the target patterns.

Interpretation: Sonification design patterns modulated by cluster feature recognition is effective, in particular in providing sounds that correspond to visualization features. The number of sound sources required may be independent of the number of agents, and a modest number of sound sources can sonify the features of large population swarms. A weakness of this method is that swarm behaviors that are not part of predetermined target patterns are not emphasized in the sonification.

## Discussion

Perceptualization is concerned with an observer and her disposition with respect to objects of study. Framing interactive sonification includes models of listening as well as models that exhibit emergent behavior. The aim is to demonstrate feasibility of extensible modeling for interactive sonification and feasibility of applying a sonficiation framework to biological information. The proposed sonification framework provides circular causality as a signal pathway for modeling a listener interacting with an experimental system that generates emergent behavior. A framework for interactive sonification is a step toward community development of a theory of sonfication, which is underdeveloped in a growing field. For example, the research area of real time EEG sonification, has seen publication growth more than 5 times (from 25 to 140) between 2002 and 2012 (Väljamäe et al., 2013).

Biological information is very likely to exhibit unpredictability such as non-linear and chaotic behavior as well as experimental system fluctuations and noise. To help disambiguate these data conditions scientific observation methods often provide numerical reference models and simulations. Adopting this approach, the sonification framework provides methods developed with models and simulations that exhibit well-known behaviors of biological systems. Working with biologically inspired simulations offers opportunity to exercise attunement with an interactive architecture addressing the entire application context including the listener's mode of interaction in the loop. This larger context accounts for observation as a secondary information system coupled to the primary data source being studied. Attunement articulates the coupling between models of observation and models exhibiting unpredictable behavior, to increase reliability in sonification applied to non-linear and chaotic systems.

In a discussion of bioinformatics Biro introduces a distinction between biological signal, data, and information (Biro, 2011). A signal emitted by a biological system is initially “data translocation” and becomes “information transmission” only when biological receptors exhibit local state changes in response to the signal. Data is transmitted throughout the system; information is transmitted only at points of responsive reception. The recipient mechanism determines what data is information. Biro's use of “information” suggests the etymology of “inform” from the Latin *informare* meaning “to give form to.”^3^ In sonification, data is formed into information when a listener is attentive to the sound and can associate it to a data source. Biro describes reception of information as a semiotic function of state change and system response. Semiotic principles provide a perspective for understanding how listeners may disambiguate sounds having representative meanings. Appendix 7 in Supplementary Material discusses related semiotic functions in sonification.

### Performance assessment of the proposed sonification framework for representing multiple experimental cases

To assess accuracy and relevance of the sonification framework, Tables 4, 5 compare three case study implementations across each of the subsystems' functional components, showing correspondence to the models illustrated in Figures 4A–C. The Case study implementations also align with the sonification framework subsystems illustrated in Figure 8, corresponding to Table 4, and Figures 10, 11 corresponding to Table 5. The framework provides a reference model for analyzing sonification implementation. For example the framework may be applied to disambiguate the model of Sound Generation for the Chua's circuit, which unlike the Swarm Chemistry sonification does not apply a separate procedural sound generator nor sonification design patterns. Instead audible sonification is generated by D-to-A conversion of the Chua's circuit oscillation. Experimental data that is converted directly to sound have been regarded as a special class of sonification, indicating the need for a more inclusive model in order to perform comparative assessment. By adopting the circuit signal as a direct sound source, the relationship of data to sound may collapse into a trivial—and ambiguous—representation. Presented in the framework component model of a sonification design, the circuit functions as a signal generator component in two subsystems, Experimental Data Source and Sound Generator (Figure 8). The circuit's dual position resolves ambiguity by referring to an underlying data acquisition model (Figure 2) that is shared in all subsystems. The shared structure of signal generator allows for two framework components to share an implementation of the circuit simulation.

**Table 4:** Sonification Framework implementation for Chua's circuit.

**Function**	**Component**	
Experimental simulation	Control space	7-dimensions: Control voltages for seven circuit components
	Phase space	Chua's circuit oscillation across seven circuit components
	Sample space	Oscillation signal at capacitor C2—a single channel of continuous frequency-amplitude oscillation
TF_1_	Chua's circuit fundamental frequency set at 50 Hz Signal extracted from component capacitor C2 routed to DAC	
Sound generator (redundant with experimental simulation subsystem)	Control space	Co-variance of control voltages of seven circuit parameters in select control regions defined in TF_3_ and inherited from the simulation control space
	Phase space	DAC of circuit signal at 44.1 kHz to an audible signal
	Sample space	Continuous audio signal of Chua's circuit oscillation is routed to loudspeaker
TF_2_	Listener acquires sound output and engages Manifold interface to explore system properties in control regions, guided by audible features	
Guided exploration	Control space	Fiducial Points for Manifold Interface based upon stable audible properties related to control space of simulation
	Phase space	Listener navigating the continuous control plane of Manifold Interface
	Sample space	Listener's cursor path in Manifold interface is output as a 2D time series data stream of (x,y) positions
TF_3_	Bijective Mapping: continuous, differentiable and bi-directional mapping transforms 2D cursor positions into multidimensional time series data of control values for seven Chua circuit components	

*Manifold Interface indicated in Guided Exploration is discussed in section Case Study 1: TF_1_ Attunement for Chua's Circuit as a Sound Generator, and in Appendix 5 in Supplementary Material*.

**Table 5:** Sonification Framework implementation for Swarm Chemistry.

**Function**	**Component**	
Experimental simulation	Control space	Total number of agents is defined as a constant; 6 control dimensions initialize agents' social behavior; heterogeneous agent types may be initialized with different control values to increase control space dimensions by 6 x *n* agent types
	Phase space	Agents' autonomous movements in a bounded 2D plane
	Sample space	Data of individual agents' movements; Statistical analysis of agents' behaviors; feature detection of phase transitions such as emergent cluster formation
TF_1_	Agent data and swarm aggregate feature data are translated by multidimensional mapping to create sound generator control data	
Sound generator	Control space	Sonification Design Patterns receive control data from swarm agent data and feature analysis data
	Phase space	Real-time sound synthesis with integration of recorded sound sources and digital signal processing
	Sample space	Sonification Design Patterns with transformations controlled by agent data
TF_2_	Listener acquires sound output and engages swarm visualization touch screen interface. Listener is attentive to explore agents' behaviors and agents' responses to perturbations guided by auditory features	
Guided exploration	Control space	Agents are visualized and displayed on a multi-touch screen. Listener's touch points in the swarm introduce “super agents” into the simulation
	Phase space	Listener observers agents' autonomous movements and determines when, where, and how to perturb agents
	Sample space	Listeners' actions applied to the multi-touch screen are transmitted to TF_3_ as 2D (x,y) positional data streams
TF_3_	Each touch position is added as a super agent to the simulation control space, with the super agent's behavior determined by the listener, not by simulation control parameters. Autonomous agents respond to a super agent as they respond to all other agents. TF_3_ enables a listener to introduce perturbations into the simulation via engagement directly with simulation Phase space	

The framework also aids comparison of case studies to identify common sonification structures having different experimental implementations. Case Studies 2 and 3 use different models of sound generation; one adopts a simple mapping of data to sound, the other applies sonification design patterns. The sonfication framework provides a structure at transfer function TF_1_ for representing attunement between experimental data and sound generation. Multiple alternative sound generator implementations can be compared at TF_1_ (see Figures 10, 11), in terms of measuring how the sound generator is tuned to the control data and how the tuning satisfies requirements of a listening scenario.

The sonfication framework provides a structure to interpret and compare attunement for Chua's Circuit and Swarm Chemistry. Table 6 identifies sonification components to define affordances based on temporality of events. At each of these components the two simulations are compared in terms of frequency measurement and frequency ratio. The degree of similarity in frequency provides quantitative comparison of how two listening scenarios are defined by temporal affordance. The selection of attunement configuration parameters in Table 6 was determined by their relevance for measuring impact of processing frequency across the two simulations.

**Table 6:** Comparison of Attunement parameters for Chua's circuit and Swarm Chemistry, based on the affordance Compatible Temporality of Events.

**Affordance Type: Compatible Temporality of Events**	**Data Type: Frequency**	
**Measurement of Attunement configuration**	**Chua**	**Swarm**
Control rate: Experimental Simulation Control Space	15–20 Hz	10 Hz
Frequency rate: Experimental Simulation Phase space	20 kHz	20 Hz
Sample rate: Experimental Simulation Sample space	44.1 kHz	12–15 Hz
Control rate: Sound Generation Control Space	15–20 Hz	20 Hz
Frequency rate of Interface update in Guided Exploration	24–30 Hz	15–30 Hz
Temporal Ratio in TF_1_: Experimental Simulation sample rate/Sound Generation control rate	1/1	1/2
Temporal Ratio in TF_3_: Guided Exploration sample rate/Experimental Simulation control rate	1.5/1–2/1	1.2/1–1.5/1

*(See Table 1, Affordances of sonification listening scenarios)*.

Table 7 provides a comprehensive comparison of the affordances that define listening scenarios for Chua's Circuit and Swarm Chemistry sonifications. Metrics and quantitative measurements can be devised for many of the fields in Table 7, including data that measures user interactions. The framework enables the impact comparison of each affordance across multiple listening scenario implementations.

**Table 7:** Listening scenario comparison of two sonification implementations using classification by affordances.

**Listening Scenario Comparison using Classification by Affordance**		
**Indicative class of users experience**	**Chua's circuit**	**Swarm chemistry**
**Affordance 1:** Sound for a domain investigation **Means:** Sound generation and sound design		
What is heard	A continuous electronic signal	Sound samples and Synthesized sounds
Range of audible change	Frequency range averages from tenor to soprano; Tone quality range from pure pitch to pure noise without pitch center; Rhythmic patterns both regular and irregular are imposed upon the frequency and tone	Sonification Design Patterns express data through multidimensional transformations including audible primitives such as frequency, duration, and timbre as well as sound events and sequences
Relation to experimental data	Sound represents oscillation dynamics and emergent properties that are signatures of non-linearity and chaos	SDP provide an aggregate audible summary of high level features; SDP are transformed by data from individual agents and groups of agents
**Affordance 2:** Interface for domain investigation **Means:** Control paradigm		
Interface presentation	Graphical user interface with cursor. Capture of cursor positions as control points. Linear potentiometers to control individual parameter values.	Graphical visualization of 2D agents autonomous movements in real-time
Degrees of freedom	2DoF as cursor moves in bounded area	4DoF afforded by two hands each having 2DoF for independent (x,y) position. Each hand position is extended by multi-finger touch constrained by limits of wrist rotation
Multidimensional control	Highly efficient Manifold interface—position of 2D cursor in a control area determines values of seven control parameters simultaneously	Touch interaction in agents' phase space provides indirect access to simulation control space. No direct modification of swarm agent control parameters is enabled for exploratory interaction
**Affordance 3:** Temporal relationship of events **Means:** Scheduling of data		
Latency from simulation control space to phase space	Very low latency—Instantaneous response	Agents' movements are updated in real time instantaneously. Emergent features evolve over extended durations of multiple seconds.
Latency across TF_1_:	Very low latency—Instantaneous response	400 ms to 2 s. Audio response to agents requires statistical analysis.
Ratio of temporal resolution of data to sound	Nearly 1 to 1	M to N. Agents' individual temporal dynamics are applied to transform an independent number of sound sources
**Affordance 4:** Multisensory information **Means:** Engaging two or more modalities of an observer		
Visible interface	GUI's discrete spatial locations in control area represent simulation control states; linear distances represent relative change of control states; marked points in control plane aid user memory of surrounding regions	Visualization of agents movements
Data visualization	None	Visualization of agents movements
Responsive interface	Audible change synchronizes with interface cursor movement	Direct touch screen interaction with visualization of agents, while listening to audible responses
**Affordance 5:** Complexification **Means:** Increasing or reducing level of detail under user control		
Ratio of simulation data to sound control data	1 to 1	1 to 1 in the first Swarm Chemistry case study. 300 to 4 in the second Swarm Chemistry case study
Reduction/compression of experimental data	Sample rate conversion of data to sound at 44.1 kHz; direct representation of data as sound	1 to 1 in the first Swarm Chemistry case study. Extensive statistical analysis applied in the second case study, reporting 5 global measures for overall agents' behavior and 4 measures for each agent cluster up to four clusters
**Affordance 6:** Learning Provision **Means:** Rehearsal for developing competence		
Access to example scenarios	Yes, an initialized Manifold interface with generating points is provided for exploration	Yes, an initialized Swarm Chemistry simulation with prepared sounds was provided for exploration
Rehearsal time given	Yes	Yes

The framework also identifies how a common function may be implemented in different subsystems. For example in both Chua's Circuit and Swarm Chemistry, attunement methods are applied to increase the reliability of sonfiying emergent behavior. In Chua's Circuit emergent behavior is tuned at transfer function TF_3_, using fiducial points located in the user interface. In Swarm Chemistry emergent behavior is tuned using automated feature recognition to control sonification design patterns, located at transfer function TF_1_.

In summary, a sonfication framework identifies structural requirements and functional components to support comparisons of multiple implementations using measurement of attributes and related affordances. An extensible framework that can be used to establish symmetry for measuring and comparing system implementations, can further be applied as a reference model for measurement and comparison of user interactions and user experiences with diverse sonification systems. Table 8 uses the sonification framework to summarize structures that align and variations in component implementation across three case studies. Structural symmetries identified in the framework can be used in the future to define human performance metrics, measuring and comparing user experience and productivity outcomes across multiple sonfication system.

**Table 8:** Summary of case studies of emergent behavior sources, attunement techniques, tuning functions, and outcomes.

	**System elements and state data**	**Attunement techniques for sonification design**	**TF_1_** **Tone production for sonification**	**TF_2_** **Listener's actuation of state changes**	**TF_3_** **Experimental data source state and transformation**	**Attunement technique and outcomes**
Chua's Circuit	Seven circuit elements that determine states & control oscillation	Explore circuit states and identify fiducial points as signature states; identify boundaries of unstable regions	System states generate oscillation data. TF_1_ applies rescaling to convert data stream to audible frequency and amplitude range.	Manifold Interface projects 7D Control Space as a 2D plane. Listener manipulates cursor in 2D to actuate 7D Control Space	User's actions modify voltage control data for 7 circuit elements, inducing oscillation state changes	Fiducial points provide stable reference points with stable auditory signatures. A set of fiducial points identify stable states to aid exploration of boundaries of unstable regions
Swarm Simulation	Autonomous 300 Social Agents moving in a bounded plane	“Literal” method: For each agent one sound is generated and the aggregate sound mixture reflects emergence	Procedural Sound generated based on [X,Y] position data of each agent, applied to frequency and stereo position of each sound	Actuation applied to system Phase Space: Listener observes a swarm simulation visualized on a 2D touch screen and interacts with the swarm by touching the swarm agents in the interface while hearing sounds.	Swarm simulation converts Listener's touch into a “Superagent” and all simulated swarm agents respond to the Listener's actions using their regular rules for agent social behavior. Swarm behaviors and patterns change as a result	Aggregating 300 sound sources as a literal sonification of agents' positions does not yield audible emergent patterns that correspond to the visible emergent patterns of agents
		Pattern recognition method: Target emergent patterns are preselected and a sound pallet is assigned to targets	Procedural Sound generated based on swarm cluster attributes and events. Preselected sound pallet is applied to salient features and transformations			Predetermined sound palette is transformed by data from pattern recognition engine; emergence of target patterns and events is translated into sound palette mix and synchronizes well with visual events

*Column one indicates the system elements that collectively exhibit emergent behavior. Column two indicates attunement techniques for sonifying emergent patterns*.

### Sonifying emergence with attunement and interactive exploration

Emergent behaviors in non-linear dynamical systems are only partially predictable in best-case scenarios. A sonification framework facing probabilistic behavior intends to provide an affordance to observe all possible states of a system. The tension between indeterminate behavior and reproducibility poses a dilemma in terms of sonification objectives:

1. Reproduce experimental system behaviors as audible signatures;
2. Render the salient features of emergent behaviors.

Restated as a methodology problem: Emergent features may not provide a data model for linear coupling to predetermined audible features. Addressing this problem, two approaches for mapping data to sound have been presented. One approach is to preselect sound to represent known features and use these audible signatures to define boundaries of unstable regions, aiding reliable exploration by close association. This is demonstrated in Case Study 1, the Chua's circuit sonificaiton using fiducial points to anchor the listener's interface with stable boundaries of unstable regions. Appendix 5 in Supplementary Material presents a related technique using attunement to compensate for hysteresis. The other approach is to render sound to represent all data points and listen for patterns in the aggregate, applying a design that aims to ensure emergent data features will generate parallel emergent audible features. This is demonstrated in Case Study 2, the Swarm Chemistry sonification using a separate sound source to represent each of 200 swarm agents. Neither approach offers a complete solution to the study of emergence. Case Study 3 demonstrates the application of sonification design patterns to provide strategies that aim to close the gap between these two approaches.

Modes of interactive exploration expand the conditions for interpreting sonfication by enhancing the listener's measure of temporal dynamics. As a listener explores a dynamical system, behaviors and sounds co-evolve. Dynamic navigation across control space provides a context to anchor sounds by association to movement and transformation. Heuristic listening involves attentive movement dynamics that complement the dynamics of control states and system responses. Modeling a listener provides criteria for measuring audible feature identification, both to assess target acquisition in known states of experimental systems, and to identify the salience of new features in emergent behaviors. Interactive exploration optimizes for emergence, with exploration supported by attunement.

### Feasibility of applying sonification attunement to data of biological signals

Future work will study feasibility of sonification attunement applied to biological signals, anticipating a two-phase approach: (1) apply the framework to sonification of a biological reference model; (2) in the sonification framework replace the reference model with a biological data source. The relative instability of biological systems presents challenges. Measurements of biological information can experience signal fluctuations introduced from an experimental apparatus. Noise may be introduced from data recording instrumentation and the surrounding environment. A biological system's states during a data acquisition trial period are unlikely to remain in a narrow mean that represents a constant value. Experimentally recorded biological data often requires disambiguation of information from noise. In line with research practices that use models and simulations as references for comparison of noisy data, sonification attunement adopts models to generate audible reference features for comparison to data that exhibits unstable system behaviors.

Physical constraints of biological systems may require specialized adaptation of the sonification framework. Feasibility study of an experimental biological system is required prior to direct application of interactive sonification. Working from a simulation to an experimental system enables comparison of workflows—of the model and the physical system—to determine symmetry between the experimental design and the simulated control configuration. Instrumentation determines where and how attunement may be applied to extend an experimental workflow. For biological experimental systems, real-time attunement feedback from tuning functions TF_2_ to TF_3_ may be challenged by physical limitations of interaction. Response characteristics of experimental biological systems may limit the capacity for real-time exploration. Time latency required to actuate state changes in a biological system may reduce the listener's sense of interaction. Establishing a parameterized exploration space for experimental acquisition of biological information requires system precision for inducing and measuring state changes. Attunement utilizes initial system exploration to identify salient features and boundaries of unstable regions. With biological information, the initial exploration process is qualified by the control parameters of the observing apparatus. Constraints in implementation of experimental control space will qualify the initial exploration of the system, which is required to identify salient features of emergent behaviors. Detecting emergence will depend upon the instrumentation and the ability to identify fiducial points in experimental control space. Finally, significant latency in experimental systems may impede interactive exploration required for heuristic listening.

### Summary of sonfication framework requirements

Sonification as data driven tone production requires interpretive and representative techniques for perceptual relevance, therefore generates design requirements. Equally, sonification requires rigor to accurately interpret and represent data or systems with reproducibility, therefore generates scientific requirements.

**Figure 13:**
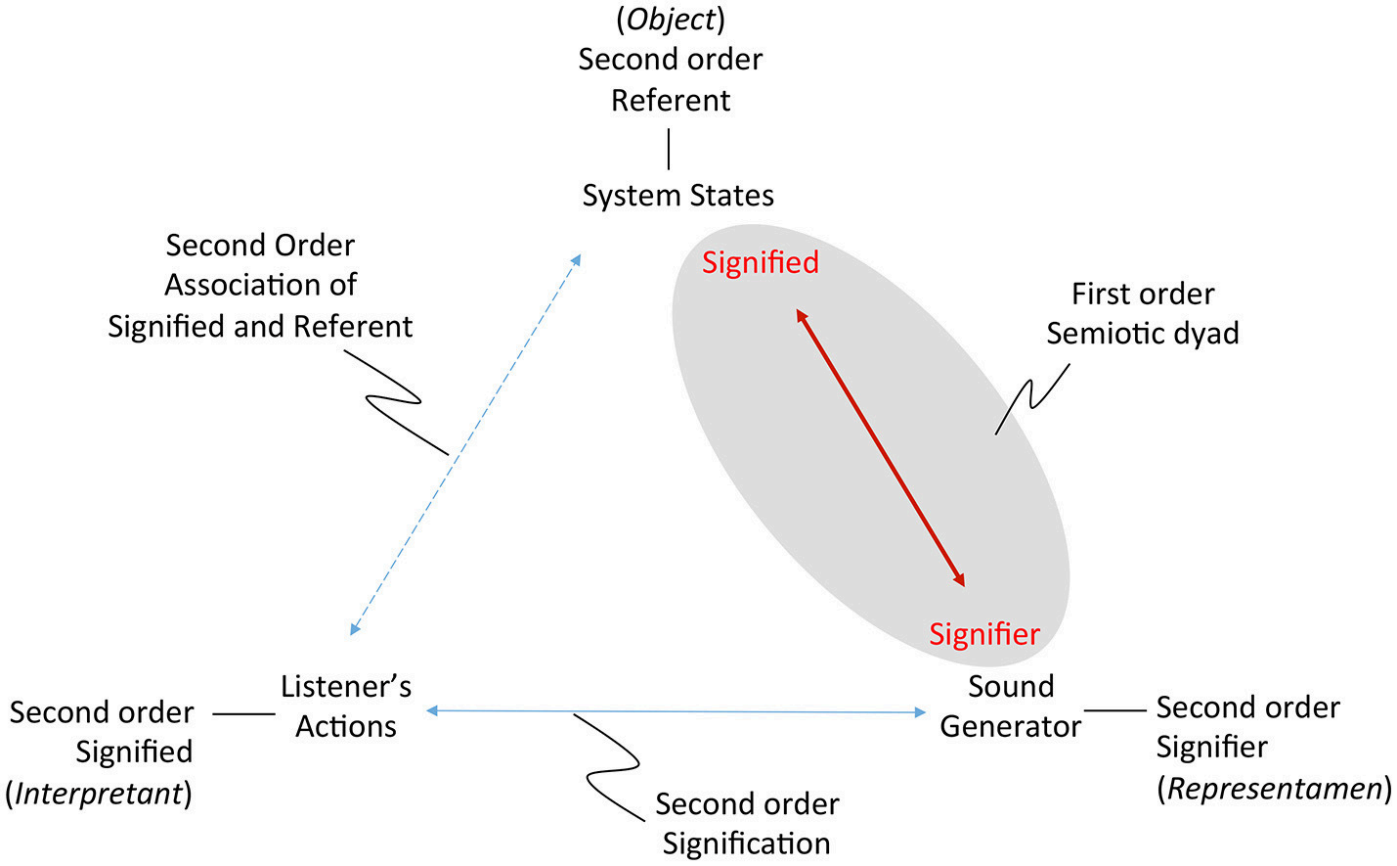
Semiotic relationships of the sonification framework. In the first-order semiotic dyad, data-driven sounds signify experimental system states. In the second-order semiotic triad, data-driven sounds signify the listener's actions that bring about the system states. The listener's actions are identified as a second-order signified. In attunement the two semiotic layers are concurrent. Terms in italics originate from Peirce's triadic semiotic model (Peirce, 1955). The vertices of the semiotic triad align with the component subsystems of the sonification framework in Figure 3.

The sonification framework provides a reference model to generate requirements for implementation of interactive data processing coupled to sound generation. The framework is designed as a canonical model of interactive sonification, providing a small number of variables to represent the model, a simple tripartite structure based on symmetry of data flow, and a reusable template that can be applied to many systems. The framework supports an attunement process to provide solutions for sonification of unpredictable data of non-linear and chaotic systems. The framework adopts a tripartite semiotic structure, which constructs the position of a sonification listener analogous to the position of a bioinformatician (Figure 13). The flexibility of the canonical model is demonstrated with two models that exhibit characteristics of biological systems, Chua's circuit and Swarm Chemistry.

To conclude, the sonification framework may be summarized as a set of requirements for design, implementation, and application of attunement.

Architecture Requirements:

1. A controllable experimental data source that exhibits emergent behavior output as a digitized signal;
2. A serial relationship of three subsystems: an active observer controlling a dynamical system, the observed dynamical system generating experimental data, and a sound generator responding to the data to generate information acquired by listening;
3. An interactive signal path that provides circular causality across the three subsystems, defining an explorable space for active listening;
  Functional Requirements:
4. An interface to vary control parameters in real time for inducing changes in the experimental data source;
5. A sound generating engine with data driven control parameters;
6. Engineered distinction between data (elicited from a signal) and information (interpretation of a received signal);
7. Three transfer functions (tuning functions) where conversion between data and information is performed for routing control signals from one subsystem to another;
  Procedural Requirements:
8. For the dynamical system that is the experimental data source, enable observers to induce changes in the system states;
9. Measure the output signals of the experimental data source to identify salient features and emergent behaviors, and transmit this information across the framework;
10. Given salient features in an output signal, enable observers to annotate the related system states by creating fiducial points in the user interface;
11. Given unstable behavior in the experimental system, identify control space boundaries of unstable regions and apply fiducial points in the user interface to mark the boundaries;
12. Apply data output by the experimental system to provide control of the sound generator, such that fiducial points have recognizable associated sounds;
  User Experience Requirements:
13. For exploring an experimental system, provide gestalt orientation for listeners to learn system behaviors through multimodal experiences;
14. For multimodal engagement, provide coupling through the experimental data source, between affordances of sound production and affordances of the user interface;
15. To generate a requisite variety of sounds, adopt sound synthesis design to generate a range of audible transformations of sounds with respect to a domain of measured changes in the experimental data;
16. To connect outputs of data pattern recognition to a semiotic function in sound, design audible signatures for known salient features of emergent behaviors.

Attunement implemented with pattern recognition provides a hybrid methodology to support reproducible observation, identification and feature discernment across multiple types of dynamic data sources using multiple types of sonification. The framework provides an efficient and extensible reference that integrates models of emergent behavior and models of a listener's attentive interaction with data. It may be applied to compare diverse sonification systems and applications, to identify common functions implemented in different subsystems, and to compare the impact of affordances across multiple implementations of listening scenarios.

## Author contributions

The author confirms being the sole contributor of this work and approved it for publication.

### Conflict of interest statement

The author declares that the research was conducted in the absence of any commercial or financial relationships that could be construed as a potential conflict of interest.

## Footnotes

^1^The concept of space is implicit in musical instruments; for example musicians' finger positions varying the lengths of strings and tubes are spatial manifestation of control structure for pitches. While varying in shapes and sizes, musical instruments commonly offer contact points and constrained space for performance navigation. The lesson to learn from this tradition is the optimization of instruments have been engineered with spatial abstraction, the distances and proportional relations for positioning physical elements; the optimization is highly targeted to achieve not only aesthetically sounding tones also physiologically coherent structure for the human body. System coherence is reflected in human capacity to explore the instrument and its audible space with facility.

^2^To demonstrate the fidelity of the simulation to the experimental system, control voltages of the physical circuit are measured and applied as control parameter values in the simulation (Zhong et al., 1994). The digital signal output of the simulation at a 44.1 kHz sample rate produces an audible signal virtually identical to that of the physical experimental circuit tuned to an audible frequency range. Extended sequences of voltage-control changes applied in parallel to the physical and simulated circuits maintain the two systems in common regions of phase space and produce audibly identical signals.

^3^http://www.etymonline.com/
