# Supplementary material for: Interactive Sonification Exploring Emergent Behavior Applying Models for Biological Information and Listening
Source: Front Neurosci. 2018 Apr 27;12:197. doi: 10.3389/fnins.2018.00197 (PMC5934483; doi:10.3389/fnins.2018.00197)
Supplement: Supplementary file 1 [file Presentation_1.pdf]

frontiers

Supplementary Material: Appendixes

Interactive Sonification Exploring Emergent Behavior Applying Models for Biological Information and Listening

Insook Choi[*]

*** Correspondence:** Studio for International Media & Technology, MediaCityUK, University of Salford, Room 2.08C, Salford M50 2HE, UK
insook@insookchoi.com

1 Appendix 1: Clinical Examples of Heuristic Listening

Clinical examples of heuristic listening can be found in auscultation, the medical practice of listening to natural sounds from the body to diagnose a patient's pulmonary, respiratory, and gastrointestinal condition (Bohadana et al. 2014). As a clinical listener, a physician performing auscultation illustrates heuristic listening in key characteristics. The primary characteristic is that sounds observed in auscultation are generated by physiological dynamical systems. Audible signatures are produced by pathological conditions, and physicians are trained using examples of these sounds as physiological indices. For example the audio signature classification nomenclature for lung auscultation includes "Stridor," "Wheeze," "Ronchus," "Fine Crackle," "Course Crackle" and "Squawk." Each class exhibits audibly distinct waveform and spectral properties in frequency band, pitch, noise components, dynamics of onset and termination, and micro-rhythmic structure (Bohadana op. cit., p.2). A second relevant characteristic of auscultation is that a patient's physiology is complex and changes over time, and sound production reflects emergent properties exhibited by the physiology. For example a Wheeze may transition from "Monophonic Wheeze" to "Polyphonic Wheeze" or the reverse, which indicates the region and area size of the affect (Pearson 2006). A third characteristic of auscultation is that signatures of sounds are non-uniform and may be mixed therefor difficult to diagnose, so in response a physician may perform guided exploration using a repertoire of patient interactions to disambiguate sounds and detect underlying conditions. Physician-guided interactions introduce dynamic local variations in physiological conditions that are tested by listening. Initially the physician will sample sounds at multiple locations on the body, and may ask the patient to change their posture or rate of respiration. Specialized interactions generate localized physiological variations. For example in lung exams the physician may prompt vocalizations having specific tonal qualities, such as Egophony – recitation of the vowel "Eeee" several times while auscultating the chest walls (Pearson op. cit. Egophony). In a healthy patient the vowel is clearly transmitted. Whereas in Bronchophony the patient recites "99" several times and the clarity of transmission increases with unhealthy consolidated lung tissue (Pearson op. cit. Bronchophony). These examples illustrate a physician performing exploratory, multisensory, interactive observations with audio sampling while modifying the physiological system states.

A precursor to the model of heuristic listening developed a music performance context to apply EEG signals for generating and controlling sound. Rosenboom applied EEG signals in music performance (Rosenboom and Number, 1997) by developing a system for data acquisition from a performer's EEG signal. Feature extraction from EEG signals used autocorrelation and other methods, applying the

measurement of listener's attention to control automated sound generation. Rosenboom's signal pathway of listening, EEG analysis and feedback for sound control anticipates aspect of sonification systems and includes a perceptual model to assess relevance of sounds. Rosenboom's description of Attention Dependent Sonic Environment (op. cit. p.74) reflects a practice in line with heuristic listening. Converse to traditional musical performance, in the EEG-based system the performer influences tendencies of evolution of a non-equilibrium instrument, as opposed to perturbation of an equilibrium instrument (op. cit. p.79). With this distinction Rosenboom implicates the sonification of nonlinear and chaotic properties, and describes conditions that are addressed using a sonification framework.

2 Appendix 2: Example of Listening Scenario Classification

Listening scenario classification is a method introduced in this research, represented in Table 1. As a test case this classification is applied to an independent study of personalized sonification of EEG feedback, published by (Mealla et al. 2014). The test case demonstrates how listening scenario classification defines the Mealla study as a partial implementation of interactive sonification. In the Mealla study, participants in neurofeedback training for relaxation were asked to select preferred intensity levels of sounds that represented data streams from their personal EEG alpha/theta signals. This selection routine addresses affordance 1 in Table 1. The listener's selection process was conducted as a dedicated task between two EEG test sessions. This selection routine addresses affordance 6 in Table 1. An easy-to-understand interface provided visual feedback of EEG and audio signals and direct control over individual sound elements, addressing affordances 2 and 4 in Table 1. Participants were presented sounds using a test EEG data stream and provided as much time as they wished to explore and select personalized settings. This routine addresses affordance 6 in Table 1. However, adjustment of sound was not available during EEG tests, indicating affordances 3 and 5 are not addressed. Results reported by Mealla et al. show that users were comfortable with the interface and that personalization improved performance in relaxation tasks. These outcomes are consistent with affordances 1, 2, 4, and 6 in Table 1. The test configuration of the reported study does not represent the sonification framework illustrated in Figure 4; it does not provide a circular causality pathway or interactive listening scenario, and participants were not able to control experimental apparatus. Still the study provides a measure of benefits for listeners developing competence through direct experience manipulating instrumentation for sonification.

3 Appendix 3: Explorable Space in the Extensible Sonification Model

To summarize the functional explorable space in the exensible sonification model: transfer functions TF_1, TF_2 and TF_3 are arranged in a circular series to transmit information and translocate data across three component subsystems. Figure 3 illustrates this serial order. Each subsystem internally implements a data acquisition model comprised of control space, signal phase space, and sample space. Figure 4 illustrates details of the implementation of this shared model in each subsystem. Each subsystem generates a signal that is internally sampled creating a multidimensional data stream to characterize the signal. Each subsystem outputs data to a tuning function where new control data is generated for the downstream subsystem. Each TF provides a tuning function for m:n mapping of multidimensional data and for isochronous differences in iteration periods between subsystems. Each TF provides a mapping from a domain of one subsystem sample space and its time interval to a range of the adjacent subsystem control space and its time interval. TF_1 receives data samples from simulation phase space and generates data for controlling sounds. TF_2 presents sounds to a listener who uses a real-time interface to generate an exploration signal for inducing changes in the

simulation. TF_3 receives sample data from the exploration signal and generates control data to modify simulation states. This series of coupled subsystems constitutes an explorable space comprising a feedback loop, such that modifications applied to any tuning function TF*n* will affect the data received and generated at the other tuning functions. Attunement is a process applied at each TF to refine the data translocation and time interval correspondence across this tightly coupled information flow.

4 Appendix 4: Sonification Design Patterns—Duration and Audibility of Data

Multiple sonification design patterns (SDP) combine to develop an audible palette. A rule of thumb is that an SDP is designed to generate a limited range of temporal dynamics. For example the minimum SDP duration may generate barely audible microstructure, providing audible building blocks for structure at greater duration; the maximum SDP duration may generate audible patterns of multiple sound events. Transitions from one SDP to another are dynamic and procedural. Experimental data is likely to exhibit salient properties at multiple timescales, requiring activation of multiple SDPs dedicated to generating audible features at different timescales. The resulting sonification is a composite auditory stream that presents discoverable features emerging from SDPs concurrently as well as in series. Audible features of SDP structure are dependent on the sound design and attributes of the sound generator. Features in SDP classes can be controlled independently at a sound generator and yet the audio signals may elicit auditory co-dependencies due to psychoacoustics of perception.

Duration perception is relative to a listener's capacity to recognize sounds and to identify sound transformations. SDP are designed to support the audio representation of data in line with the multi-temporal structure of listening presented in section 2.3. SDP cannot control two listener-based dependencies: 1) the changing duration of neural processing when pre-attentional sounds become attentional, and 2) the potential for perceptual formation of macro-sound patterns (>1sec) from micro sounds (<200msec). Both dependencies are related to the dynamic multi-temporal structure of audibility, characterised by (Teng et al. 2016, p.1):

> Natural sounds contain information on multiple timescales, so the auditory system must analyze and integrate acoustic information on those different scales to extract behaviorally relevant information… The temporal integration of sound is a fundamental property of hearing. Integrating information over time…serves as a basic information accumulation process in the auditory system to guarantee that enough information is supplied for extracting meaningful regularities on a certain timescale. …(E)vidence suggest(s) that the auditory system extracts fine-detail acoustic information using short temporal windows and uses long temporal windows to abstract global acoustic patterns.… These findings support the hypothesis of a dual-scale processing likely implemented in the auditory cortex. …(T)hat temporal integration involves a process of sampling or integrating information discretely on a small timescale (e.g, tens of milliseconds) and on a large timescale (e.g. hundreds of milliseconds) *instead of simply integrating information continuously* (emphasis added).

SDP can be used to control multi-temporal structure, from micro sound variation to the formation of macro sound patterns. The italics in the above quote from Teng indicate that SDP may also generate separate parallel patterns at different timescales. The SDP duration over which a listener accumulates information is asynchronous to when she extracts meanings. Not all micro sounds are hierarchically captured in larger percepts; research demonstrates that depending on the listening task micro-duration sounds may compete for attention against long duration sounds (Teng op. cit.; McDermott et al.

2013; Fritz op. cit.). Teng et al. (op.cit.) identify <50ms as the local fine-grained information processing timescale, and concurrently, >200ms as the global timescale for pattern extraction. This evidence suggests that SDP structure can be optimized by study of the simulation data and experimental apparatus to determine the properties most relevant for sonification, then aiming for compatible temporal dynamics in the selection of sound generators. Listeners' recognition of structures greater than 200ms develops during the accumulation and statistical summary of percepts; this accumulation period is concurrent with percept transition from pre-attention to attention (McDermott op. cit.). Piazza et al. (op. cit.) found structures greater than 3 seconds emerging in audible ensemble coding (section 2.3). These durations are compatible with the SDP attribute controls in Table 2. Similar classifications of audible features have been applied in studies of listeners' EEG responses to audible sound transformations (Lin et al. 2014).

SDP support Heuristic Listening for Temporal Evolution Temporal evolution is a common characteristic of dynamical systems and sound is a compatible medium to represent patterns evolving in time. Compatibility is based on perceptual pairing between two time domain systems, one inaudible and one audible. Observers may intuit behaviors of a dynamical system from behaviors of sound qualities, however the pairing of two time domain systems cannot rely upon sound qualities inherent to the observed system. A dynamical system's salient features are not necessarily discernable with appeal to human auditory perception, even when the experimental data may be presented in an audible signal (Hermann 2011). Artefacts are generated when data is converted into sound; the aim of sonification is to determine the nature of those artefacts and maximize audibility of salient features originating in the data. SDP can be applied to coordinate transformations of multiple audible attributes at multiple timescales, organized to maintain coherence of sound sources and transformations. Model-based data-driven interactive sound for VR was introduced by (Bargar et al. 1994) and model-based sound generation has been applied to interactive multimodal performance by Choi (Choi 2000b) and also applied to sonification by Hermann (op. cit.).

To establish a basis for coherent sound generation SDP may be designed by consulting the model of heuristic listening in an interactive listening scenario. The heuristic listening model is implemented and encoded in SDP. In this approach an SDP takes into account the characteristics of the interactive control structure applied to the experimental system. Interactive exploration in the sonification framework determines properties of transitions in the experimental simulation, which in turn influence the data properties that modulate SDP. In other words, the full integration of SDP originates with heuristic listening and the listener's supramodal attentional mechanisms at the Guided Exploration subsystem, and extends across TF_3 continuing to TF_1 output.

5 Appendix 5: Manifold Interface Applied to Fiducial Point Tuning for Structured User Interaction with a High Dimensional System

Covariance of seven circuit elements is required for agile navigation of nonlinear phase space. (Choi 2000a) introduces a *manifold interface* technique for interactive covariance for *n*-dimensional control parameters. The manifold interface provides a general solution to continuous linear covariance of 3 or more control parameters by representing the *n*-dimensional control space as a bounded plane. Fiducial points of *n*-dimensional control space are represented as two-dimensional points located in a rectilinear GUI interface area. Navigating a cursor in the interface area generates a stream of output values in *n*-dimensional control space. Between and around fiducial points the 2D manifold surface interpolates values to generate *n*-dimensional outputs. To implement this interface a geometric method applies a transfer function in a bounded subregion of *n*-space that is continuous,

differentiable and bi-directional between the two-dimensional interface area and the seven-dimensional circuit control space. TF_3 applies the manifold interface to transform user's 2D movements at the interface into a series of control voltages for seven circuit elements. Figure 12 provides a schematic illustration of a path in *n*-dimensional parameter control space where $n > 3$ dimensions are projected as a two-dimensional control path on an interface plane. In Figure 12 the four lines projected between spaces indicate the positions of four fiducial points in control space and in actuation space. 2D points and the interface area orient listeners for compatible exploration.

The manifold interface is an example of an optimized explorable space integrating attunement techniques. Techniques include the selection of fiducial points and the arrangement of fiducial points in the interface plane, to create regions between points for exploring nonlinear dynamics while avoiding limit cycles and fixed points (section 4.1.1). Selecting fiducial points is part of the attunement process, defining correspondence between salient features of the sound, selected circuit states, and positions of fiducial points in the interface.

Using Fiducial Points to Enhance Empirical Observation Fiducial points are multidimensional control states, created based on empirical observation of control states that produce characteristic oscillations with high probability. In the attunement model for Chua's circuit, TF_2 is an arrangement of a set of fiducial points that configure corresponding control states for seven circuit components. The attunement process identifies fiducial points in multiple regions of control space with respect to characteristic oscillatory behavior in each region. The oscillation may be periodic, quasi-periodic, intermittent, or chaotic; a high probability of reproducibility of any of these may be selected as a fiducial point.

Listeners' exploration of Chua's circuit involves traversing the control space between fiducial points. The exploratory interface between TF_2 and TF_3 enables a listener to covary the control voltages of the seven circuit elements (Section 4.1.1). In the continuous control plane of the manifold interface, modifying the distances between fiducial points in the interface optimizes the resolution of control space regions accessible between the points. Greater distance between two points refines the level of control by decreasing the step-size of control increments in the output signal of the interface. Distances and arrangements of fiducial points' positions in the interface effectively determine a listener's access to control space and provide constraints for inducing state changes. Arranging fiducial points is an example of attunement to aid listeners' exploration of boundary conditions and phase transitions while avoiding both ultra-stable and undesirable regions.

Adapting Attunement to Sonify Hysteresis of Chua's Circuit In regions of control space that exhibit hysteresis a given set of control values do not reliably reproduce a consistent oscillation. When a control state exhibits hysteresis a related fiducial point will be inconsistent in the phase space it generates. To generate reliable sonification in unstable regions, fiducial points are identified in bounding regions. To establish these boundaries the system is explored to identify control space regions that exhibit hysteresis and boundaries of these regions. Fiducial points are selected where oscillations exhibit stable states and distinctive features. The attunement method detailed in section 3.2 is applied to convert fiducial points into interface generating points (GP). In the manifold interface GP are arranged at regular intervals such that unstable control regions are surrounded by stable control points. The interface layout of GP facilitates exploration by planning control paths through unstable regions, departing from and returning to stable regions. distances between GP provide orientation for listeners to explore the relative distribution and adjacency of regions that are stable, transitional and unstable. Rate of change as well as the direction of the control path from one region to another helps characterize the onset of hysteresis. A control path defined along the edge of

a hysteretic region helps to differentiate the conditions for hysteresis as a combination of three factors: 1) the values in control space, 2) the sequence of control state changes, and 3) the rate of change of control state.

6 Appendix 6: Observer's Social Interaction with Simulated Autonomous Agents

In the Swarm Chemistry simulation, TF_3 is implemented by conveying touch data from the display surface to the control space of the agent simulation. A *super agent* introduced at each touch point enables agents in the simulation to respond to listener's actions. Listeners can influence agents through actions such as dividing a cluster, gathering and merging clusters, or guiding agents toward or away from other agents. An implementation with a touch-screen interface is reported in (Choi 2017a)

The swarm phase space is comprised of agents' collective states and positions. Interaction in phase space is local and instantaneously introduces a super agent while preserving continuity of automonous agents' responses. Super agents are introduced and deleted in real-time processing without creating discontinuity in the state space model. A listener's interactions are introduced into phase space in the form of one or more super agents, with movements determined by listener's actions. While a super agent moves independently the autonomous agents respond as if to a normal agent. Freedom of movement empowers the listener to exert influence through social interactions that are probabilistic, as the other agents continue to move and respond according to autonomous tendencies. In this way interface control of the swarm is an emergent property of the simulation, and as such is indirect and indeterminate. Using super agents a listener cannot directly manipulate other agents; super agents can only exert social influence in the form of proximity, speed, and movement direction. In this sense the listener must use super agents to socialize with other agents in order to influence behaviors. Super agents' independence includes persistence—the ability to remain in a fixed position across multiple times steps, whereas autonomous agents move at every time step. Super agents' independent movement and persistence of position enable a listener to prolong autonomous agents' collective formations, to induce phase transitions and to avoid phase transitions.

7 Appendix 7: Attunement as a Dynamic Semiotic Model for Heuristic Listening

Models of listening provide criteria for determining the formation of meaningful sounds. To consider the formation of meaning, a semiotic model for heuristic listening is introduced with respect to biological information transmission. Biro (section 1) describes bioinformatic signal reception as a semiotic function of state change and system response. Sonification adopts a signal path similar to that described by Biro for bioinformatics. Semiotics can be traced to functions where data mapping is performed. In the sonification framework the design of tuning functions TF_n requires mapping techniques. In semiotic terms a mapping is a dyad of signifier and signified, a sound signifying a data source. A semiotic model can explain how sounds acquire meaning in sonification, but does not account for implementation with nonlinear and unstable data sources. With emergent properties such as hysteresis we observe that one-to-one correspondence between a data source and a sound is inconsistent. The inconsistency of correspondence creates semiotic uncertainty – an audible signature does not consistently refer to a singular system state. Attunement addresses this inconsistency with a method to develop reliable indicators at boundaries of unstable data regions; by exploring boundaries a listener remembers differences. An example is found in chaotic systems that exhibit recurrence (see section 4.1.1.); signal trajectories in chaotic phase space tend to visit recurrent points more frequently, and densities of recurrent points form invariants that provide stable data with respect to

immediately surrounding regions (Faure and Korn 2001, p. 784). Sonification of recurrence regions selected as fiducial points provides stable audible signatures that return with reliable frequency as the listener explores boundaries. A semiotic model that incorporates dynamics is not a mapping from data to sound but a relationship from actions generating data to sound.

A listener's exploratory process of discovery and auditory memory can be described using a triadic semiotic model rather than a dyadic model. A triadic model formalizes a listener's cognitive function separately from signifier and signified: a listener's auditory memory contributes to concepts independent of a predetermined mapping. In response to data (a phenomenon) connected to sound (signifier of the phenomenon), a listener develops a concept of an audible control region (a listener's expectation of the phenomenon). The concept is not merely a cognitive response to a semiotic stimulus, it is a listener's predictive disposition entrained by a semiotic context. Predictive disposition is a strong function in heuristic listening (section 2.4.1), engaged with interactive exploration through the sonification framework.

Attunement is compatible with a dynamic semiotic model providing a triadic structure for heuristic listening. Figure 13 illustrates the relationship between a semiotic dyad for sonification, and a semiotic triad for interactive exploration. The dyadic semiotic model of data-to-sound is embedded in the triadic model. In the dyad, sonification signifies data; in the triad, sonification signifies the listeners' engagement with the system that generates the data. The dyad is considered first-order semiotics and the triad represents a second-order semiotics that reflects awareness of the context that defines the dyad. Figure 13 introduces classifications from C. S. Peirce's triadic model of semiotics (Peirce 1955). The triad is formed by the *object*—a phenomenon that information refers to; the *representamen*—the form in which the referring information is observed, and the *interpretant*—how an observer makes sense of the referring information with respect to the object. Figure 13 aligns Peirce's semiotic triad with subsystems of the sonification framework in Figure 3. The *object* aligns with the experimental system states, the phenomenon to be signified. The *representamen* aligns with the sounds, the referring information observed. The *interpretant* aligns with the listener's actions at the interface, the listener's dynamic conceptualisation of the simulated system through the interactive interface. The listener's actuation, the *interpretant* is a second order signified. Second order semiotics account for concept formation by the observer. An interpretant embodies an observer's conceptualization of the semiotic process.

Second Order Listening Attunement implements a model of second order semiotics, and heuristic listening can be described as second order listening with awareness of the sonification framework. In terms of the attunement process, the second order signifier describes a relationship between control space dynamics and phase space dynamics. This is observable as "dynamics of dynamics," revealed by interaction through exploration across control space. A first order signified occurs when a listener hears stable dynamics of phase space in an unperturbed control state. A second order signified occurs when a listener hears evolving changes in dynamics of phase space induced by exploration of control space. By exploration a listener can differentiate the signatures of two types of dynamics and recognize potential boundary regions where changes occur in the audible quality of the phase space dynamics. Exploring the control space of the system reveals the temporal signature of how the dynamics change for different system states. The temporal signature of the triadic semiotic model describes a mode of attentive listening that can be supported by attunement to achieve reproducible sonification for unpredictable data.

Correspondence with Bioinformatics Biro (op. cit.) uses a triadic model to describe bioinformatics, identifying a second order semiotic function in the observation of biological information. His

description is roughly analogous to the semiotic model of the sonification framework illustrated in Figure 13. Biro introduces a triadic model to represent the roles of bioinformatician, biological subject, and bioinformatics data. For Biro a bioinformatician (Peirce's *interpretant*) is an external observer who identifies transmission of biological information under two conditions: 1) non-random data flows from a subsystem of a biological organism (Peirce's *object*), and 2) another subsystem responds to the data with internal state changes (Peirce's *representamen*). Biro's model asserts that biological signals function as *data translocation* rather than as information transmission. In a translocation model, biological data does not transmit information except when received at another subsystems responding with state change.

Characteristics of Biro's bioinformatics model are reflected in the sonification framework. Data of unique type is generated in each subsystem. Tuning functions do not transmit data from one subsystem to another. A tuning function translocates data of one subsystem, and a mapping enables another subsystem to respond by changing states, generating analogous internal data of its own type.
